# Supplementary figures and images for: Whole-genome sequencing and gene sharing network analysis powered by machine learning identifies antibiotic resistance sharing between animals, humans and environment in livestock farming
Source: PLoS Comput Biol. 2022 Mar 25;18(3):e1010018. doi: 10.1371/journal.pcbi.1010018 (PMC8986120; doi:10.1371/journal.pcbi.1010018)

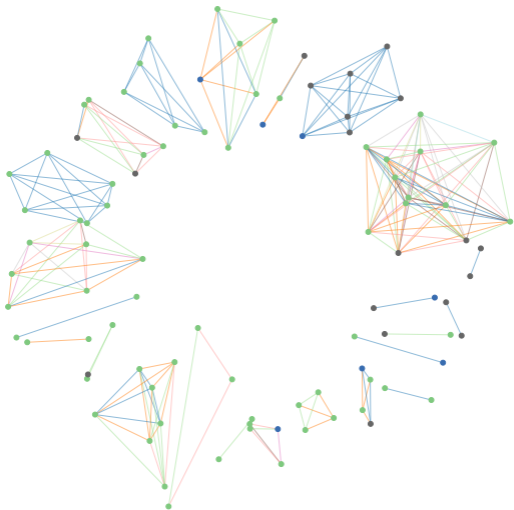

■ Chicken  
■ Environment  
■ Human

— 0.0  
— 1.0  
— 2.0

— 3.0  
— 4.0  
— 5.0

— 6.0  
— 7.0  
— 8.0

Supplement: S1 Fig — Network diagram showing pairwise connections between human, chicken and environmental isolates with less than 15 pairwise SNP differences. The lines between pairs of isolates are colour-coded by SNP number. (PDF) [file pcbi.1010018.s001.pdf]

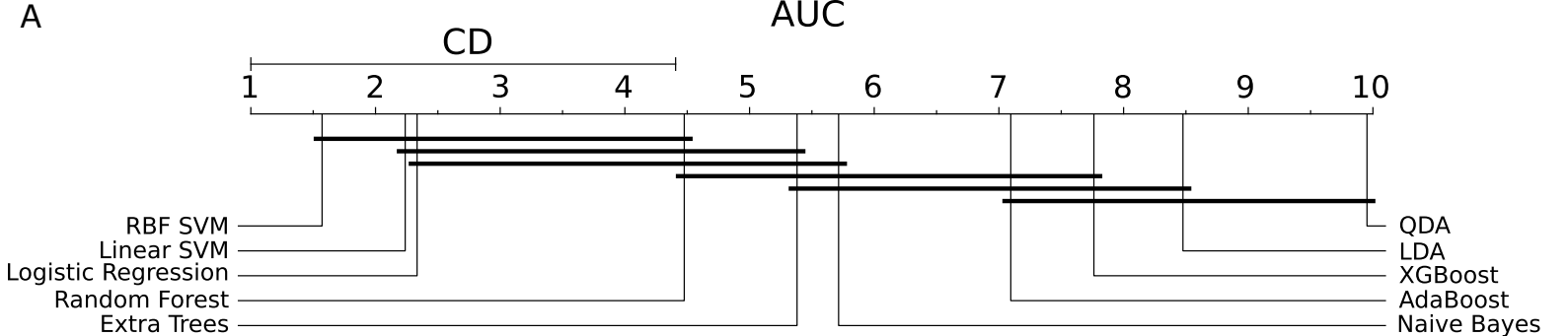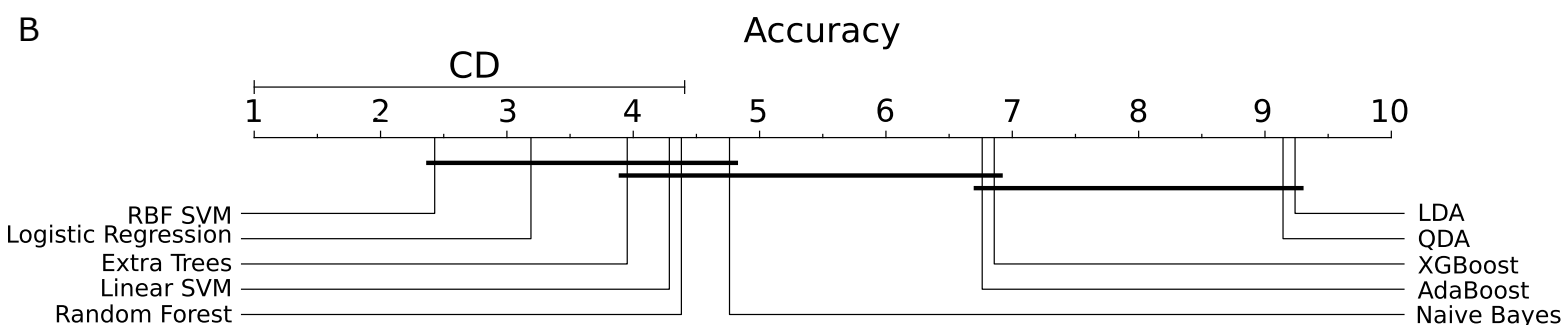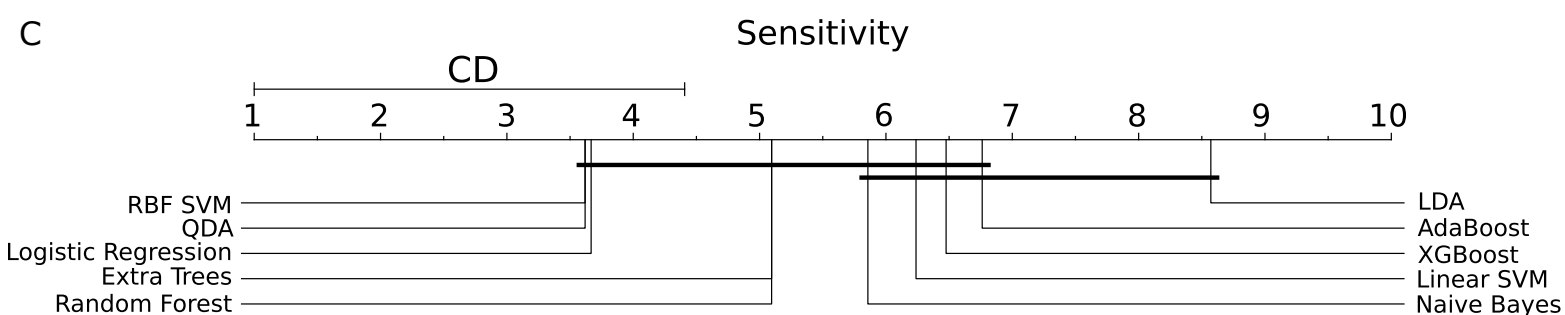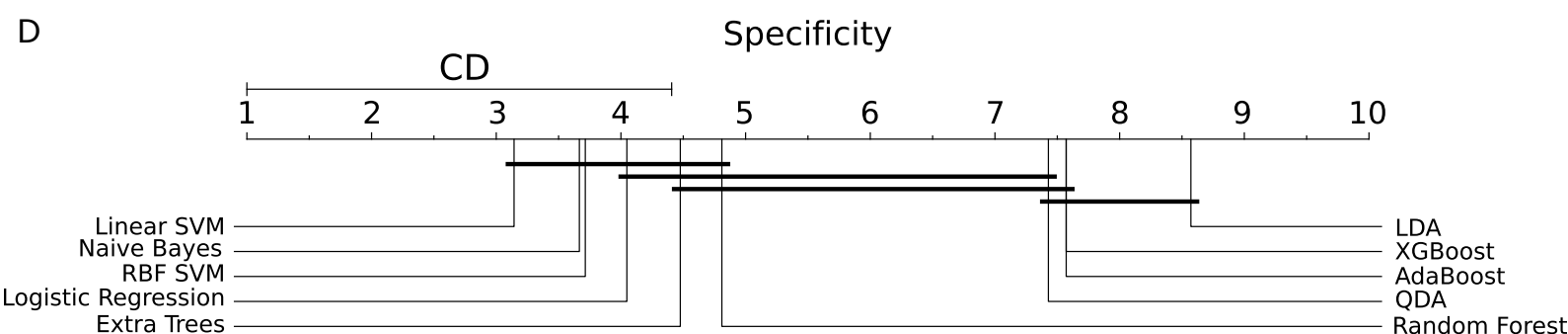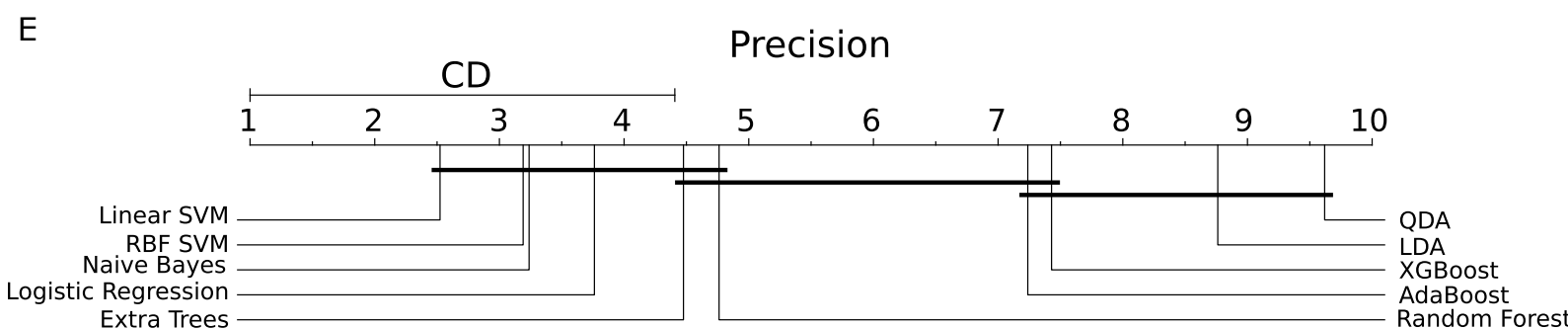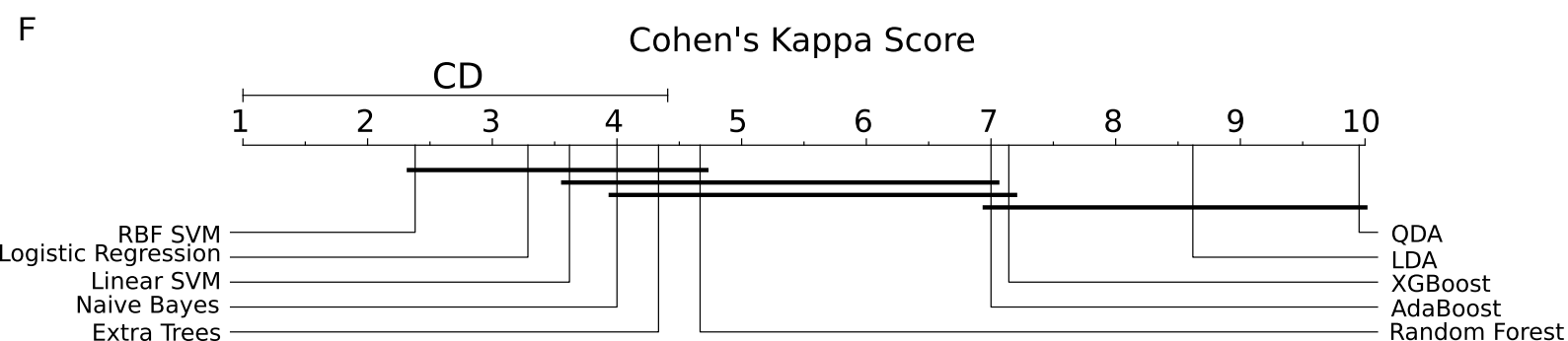

Supplement: S2 Fig — Nemenyi post-hoc test for the performance metrics (A) AUC, (B) accuracy, (C) sensitivity, (D) specificity, (E) precision and (F) Cohen’s Kappa score for the following classifiers: logistic regression, linear SVM, RBF-SVM, extra tree classifier, random forest, adaboost, xgboost, naïve bayes, linear discriminant analysis (LDA) and quadratic discriminant analysis (QDA). (PDF) [file pcbi.1010018.s002.pdf]

**A**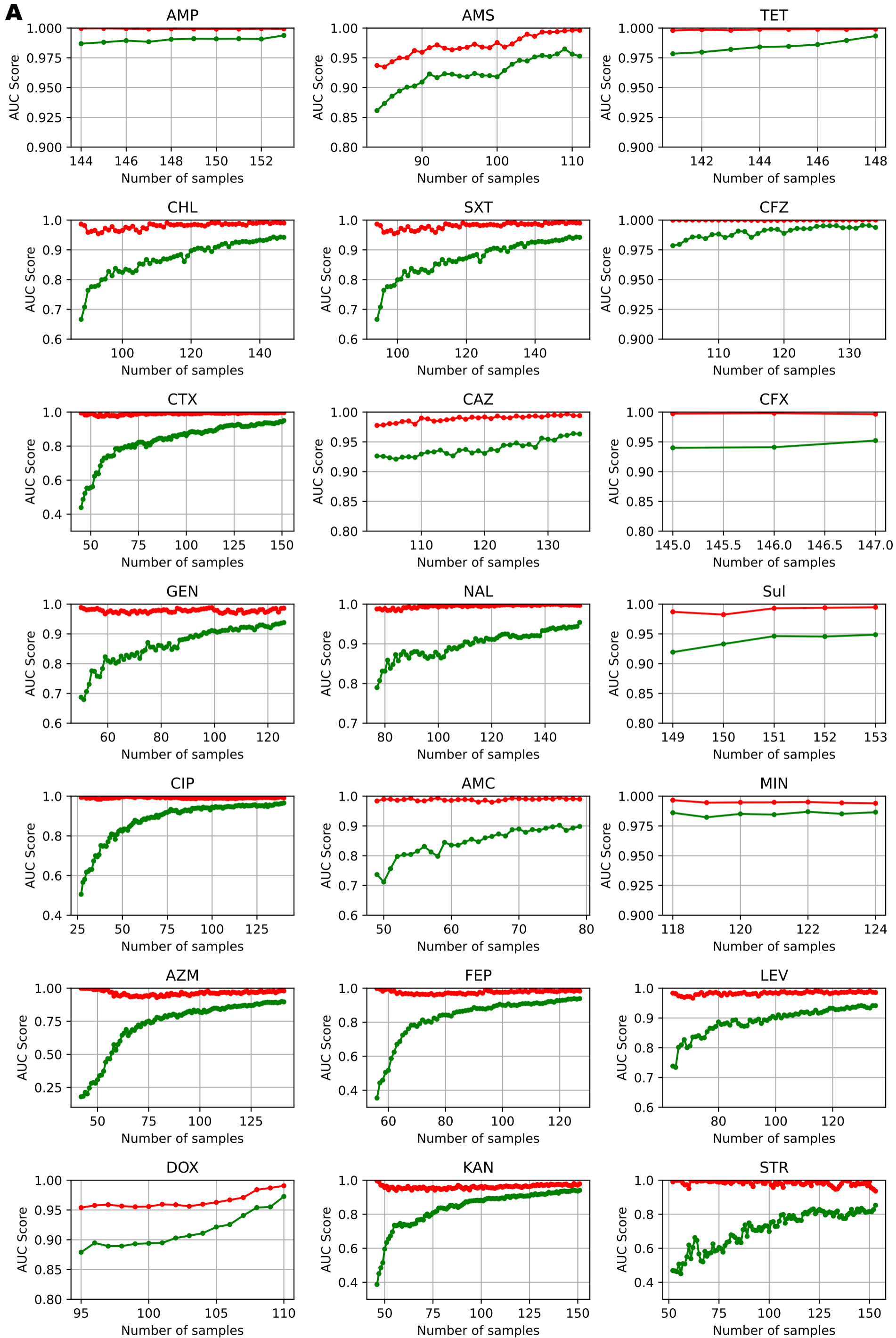

**B**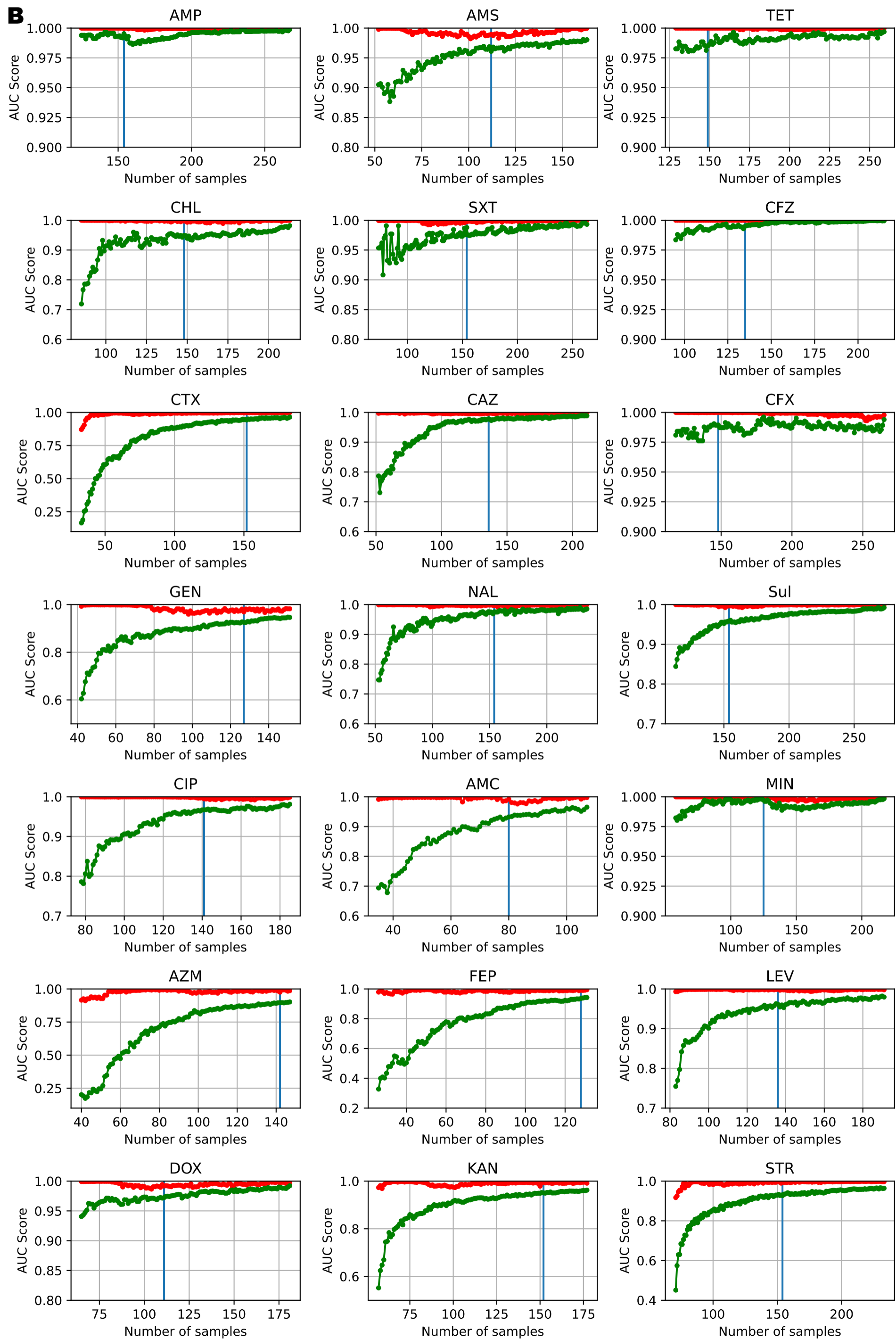

Supplement: S3 Fig — Learning curves of the 21 antibiotics using a wrapper backward selection to evaluate the training and testing performance whilst decreasing the number of samples. (A) The original number of samples for each antibiotic and 5 nested cross validation iterations with an RBF-SVM as the main classifier; (B) SMOTE approach as a pre-processing step to increase the number of samples by adding synthetic samples to the minority class for each antibiotic and 5 nested cross validation iterations with an RBF-SVM as the main classifier. In both cases, the same features used to acquire the performances in the Fig 2 and S2 Table are used and kept for all the iterations of the WBS. The red lines indicate the training performance, the green lines the testing performance and the blue vertical line the original number of samples. (PDF) [file pcbi.1010018.s003.pdf]

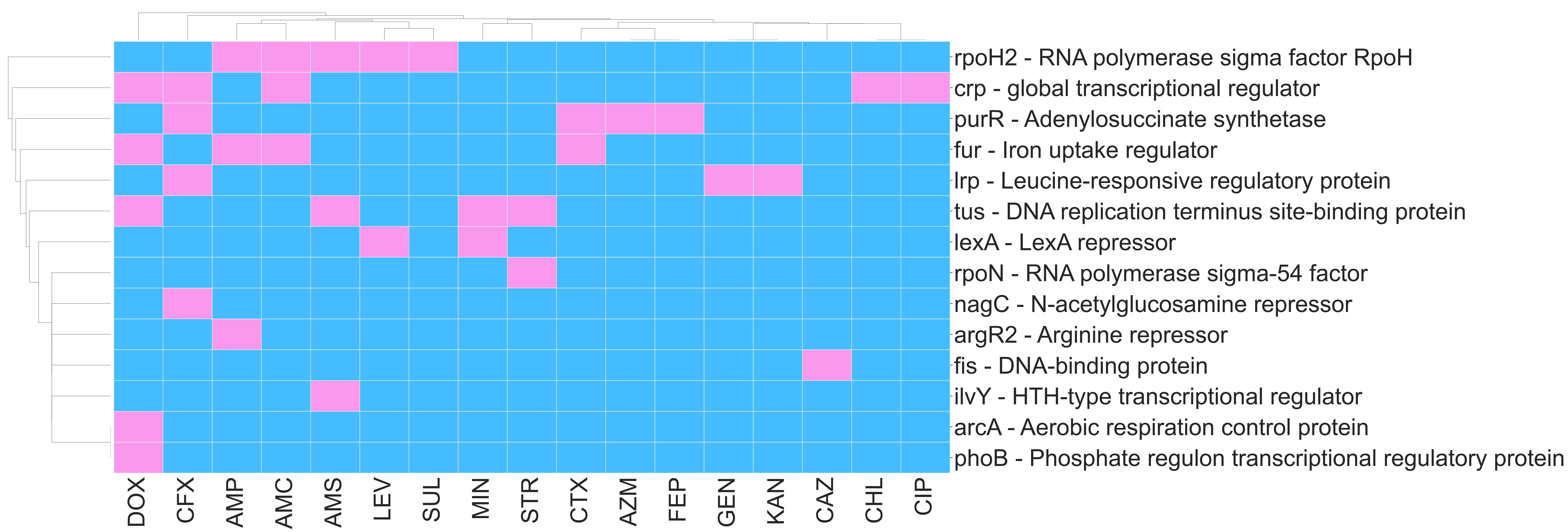

Supplement: S5 Fig — The heatmap shows for each antibiotic model, transcription factors where the k-mers selected by machine learning mapped to binding sites for the transcription factor and the proportion of individual isolates hit were more than 30% different between the resistant and susceptible isolates. The presence of a binding site hit is indicated in pink, with blue denoting no hit. Antibiotic models with no significant hits were neglected from the figure. (PDF) [file pcbi.1010018.s005.pdf]

**(A)**

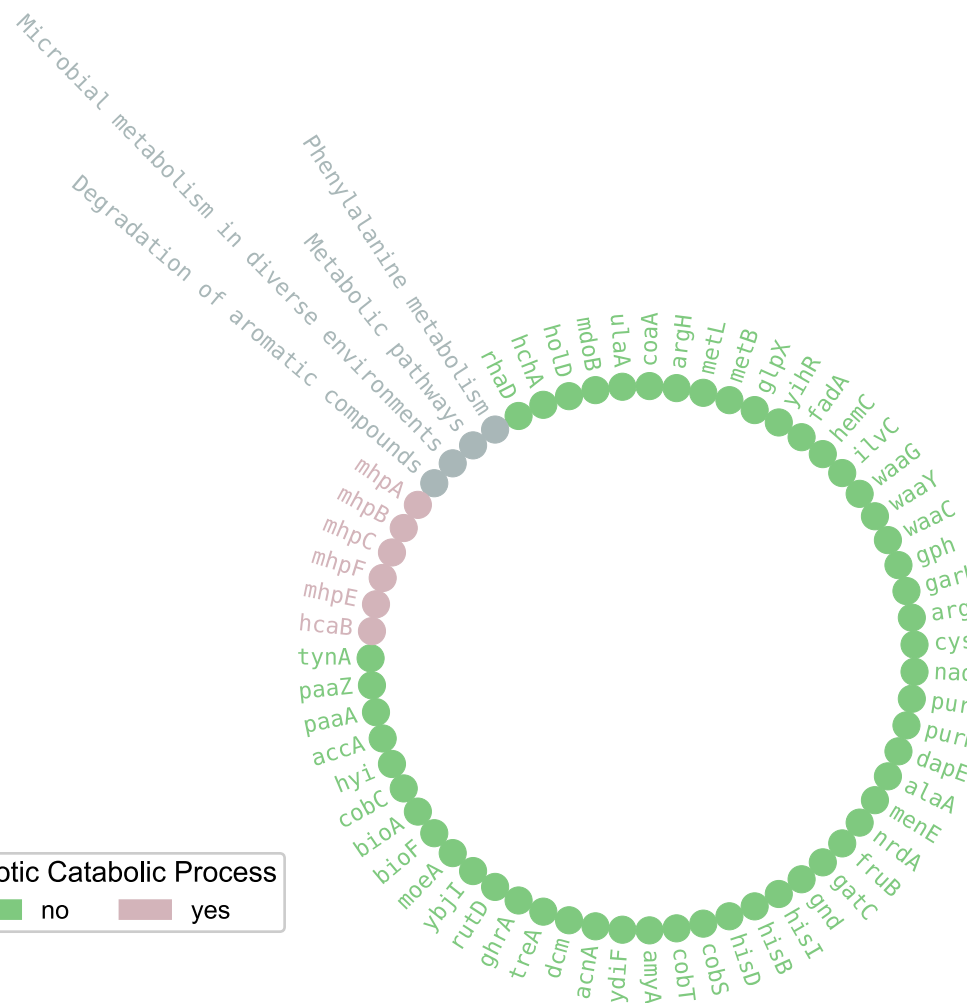

**(B)**

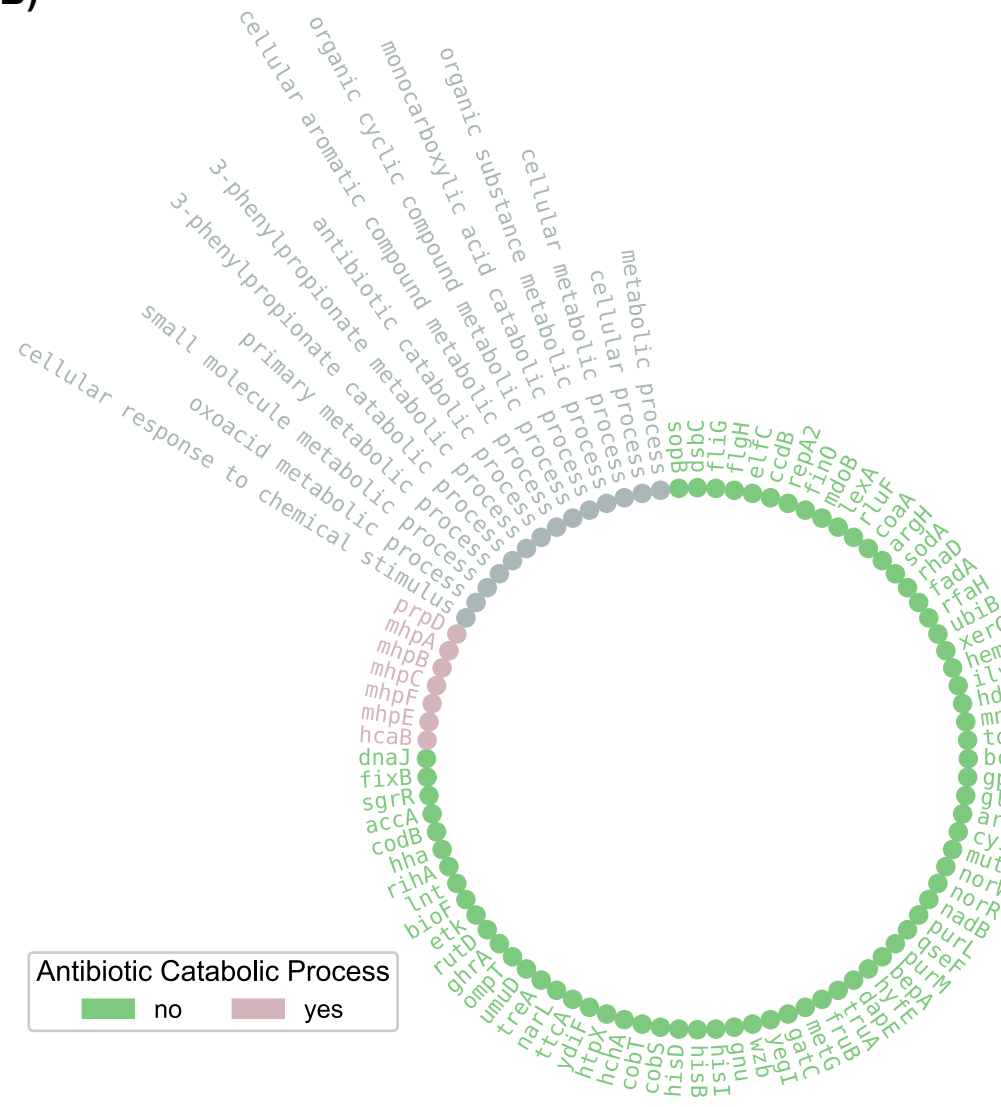

Supplement: S6 Fig — (A) Circos plot of KEGG pathways found to be significantly enriched in the 278 unique genes found by the machine learning compared to the whole genome background not annotated as ARM in public databases. (B) Circos plot of gene ontology molecular functions found to be significantly enriched in the 278 unique genes found by the machine learning compared to the whole genome background not annotated as AMR in public databases. (PDF) [file pcbi.1010018.s006.pdf]

(A)

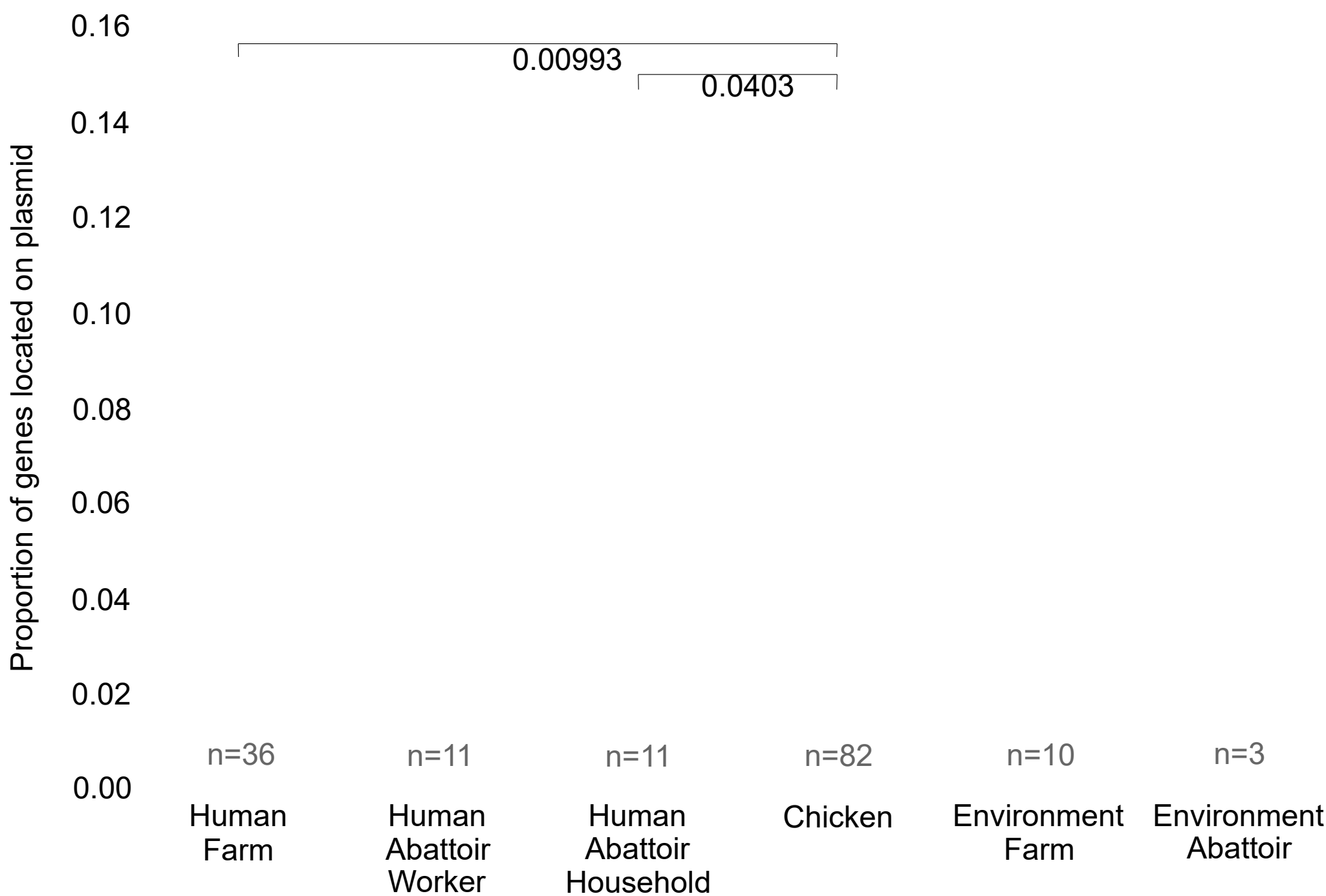

(B)

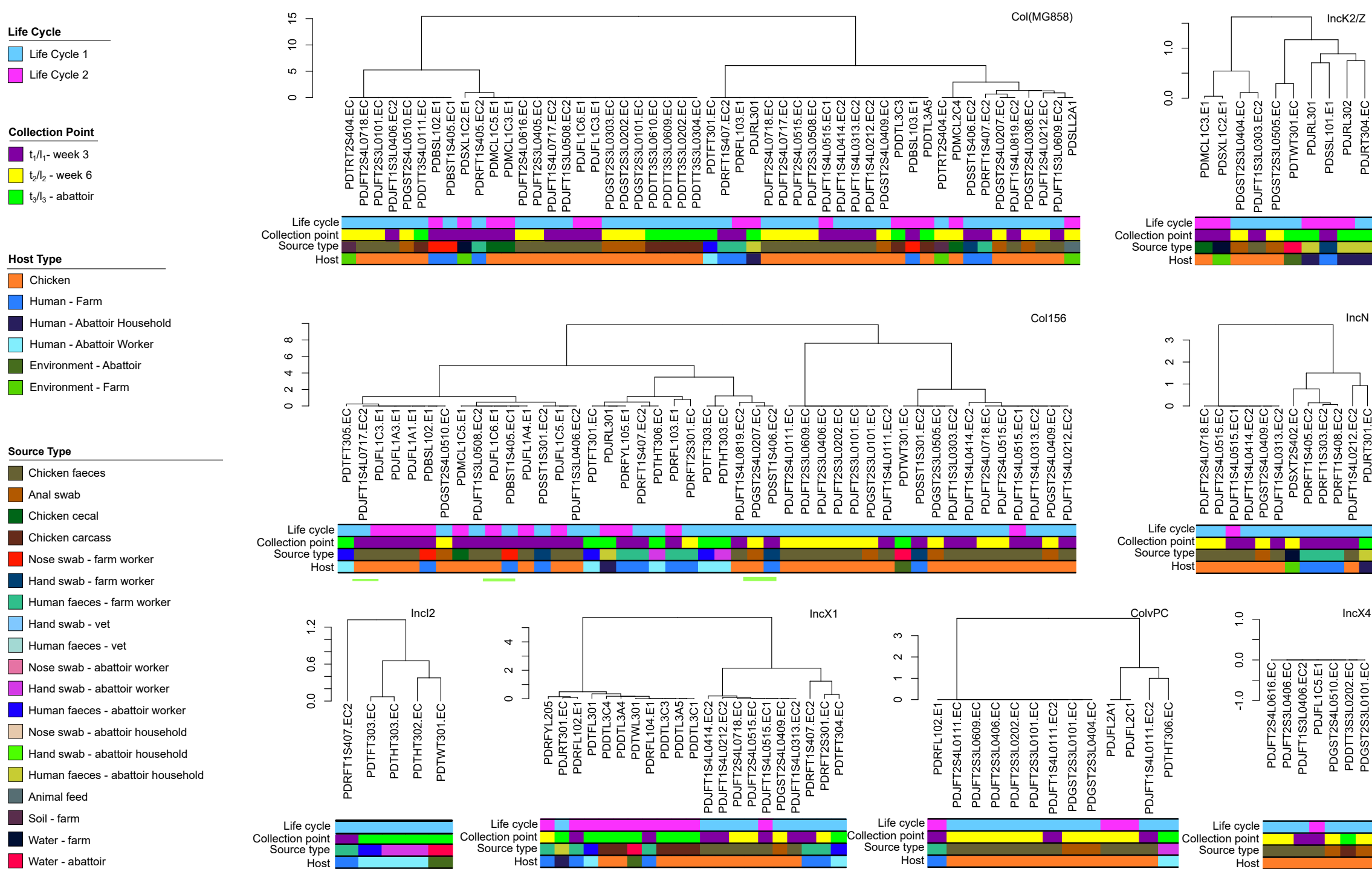

Supplement: S7 Fig — (A) Boxplot showing the proportion of genes located on plasmids per isolate grouped by host type. Isolates from chicken were found to have significantly more genes located on plasmids that human farmworkers and abattoir worker household members. (B) Hierarchical clustering of plasmids with low number of significant genes grouped by replicon type. Clustering was based on gene presence or absence. Green underscores indicate areas of high similarity (>0.996 WGS ANI value) between isolates from different hosts, suggestive of transmission between hosts. (PDF) [file pcbi.1010018.s007.pdf]
