# Supplementary material for: Whole-genome sequencing and gene sharing network analysis powered by machine learning identifies antibiotic resistance sharing between animals, humans and environment in livestock farming
Source: PLoS Comput Biol. 2022 Mar 25;18(3):e1010018. doi: 10.1371/journal.pcbi.1010018 (PMC8986120; doi:10.1371/journal.pcbi.1010018)

A. Amoxycilin/clavulanic acid (i) (ii)

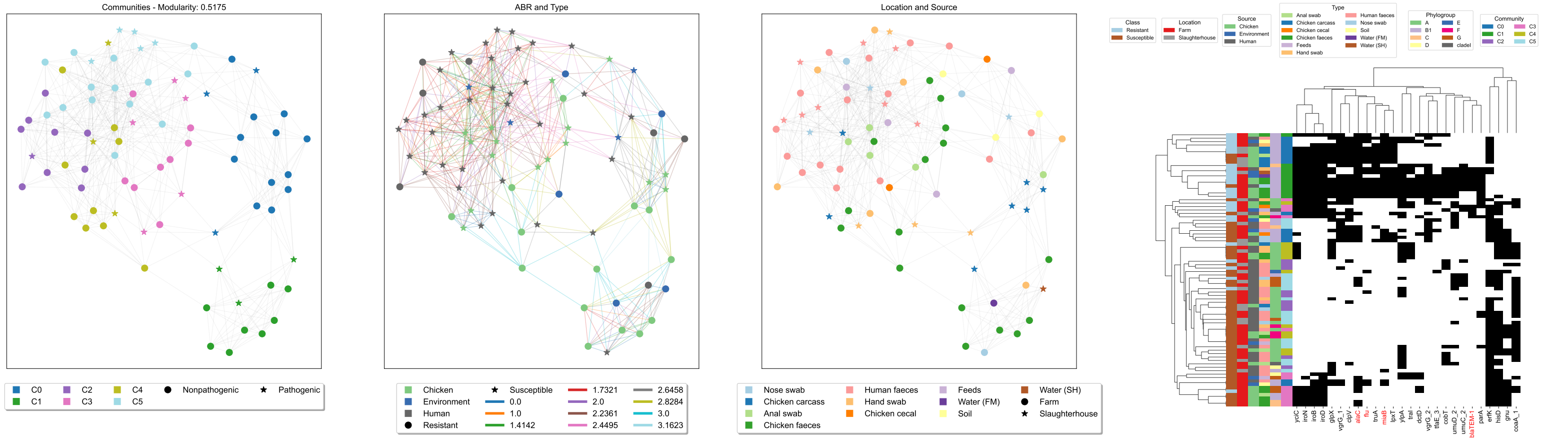

B. Ampicillin (i) (ii)

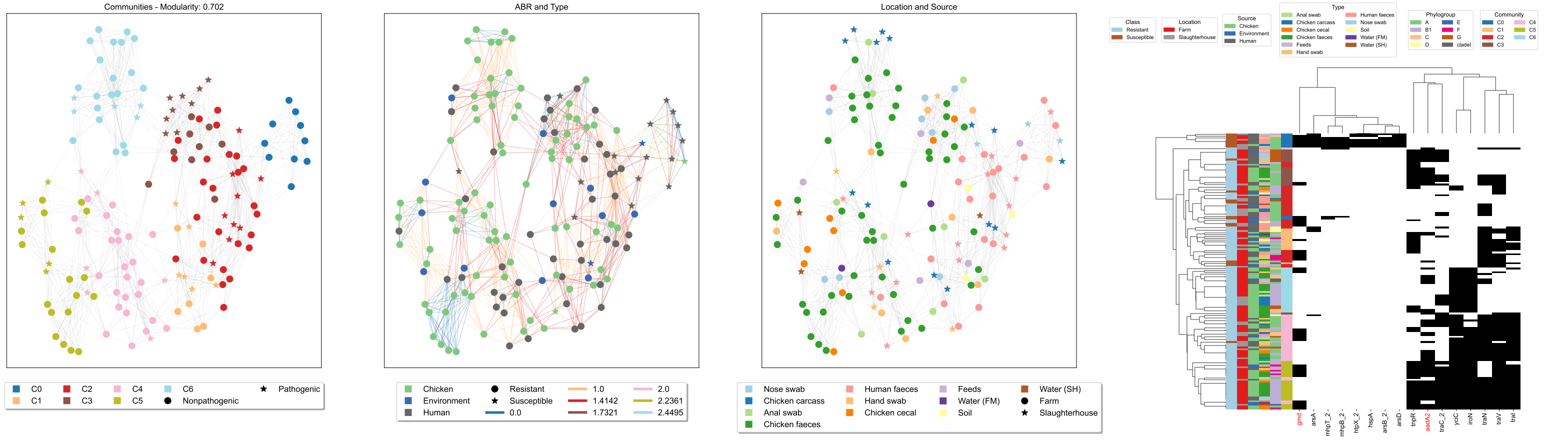

C. Aztreonam (i) (ii)

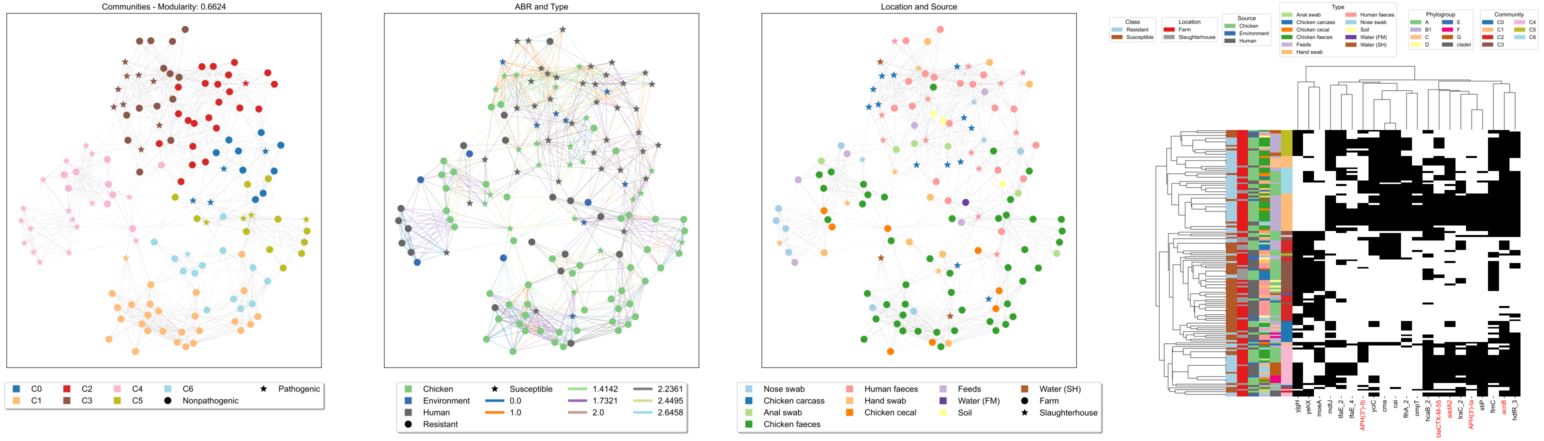

D. Ceftadizme (i)

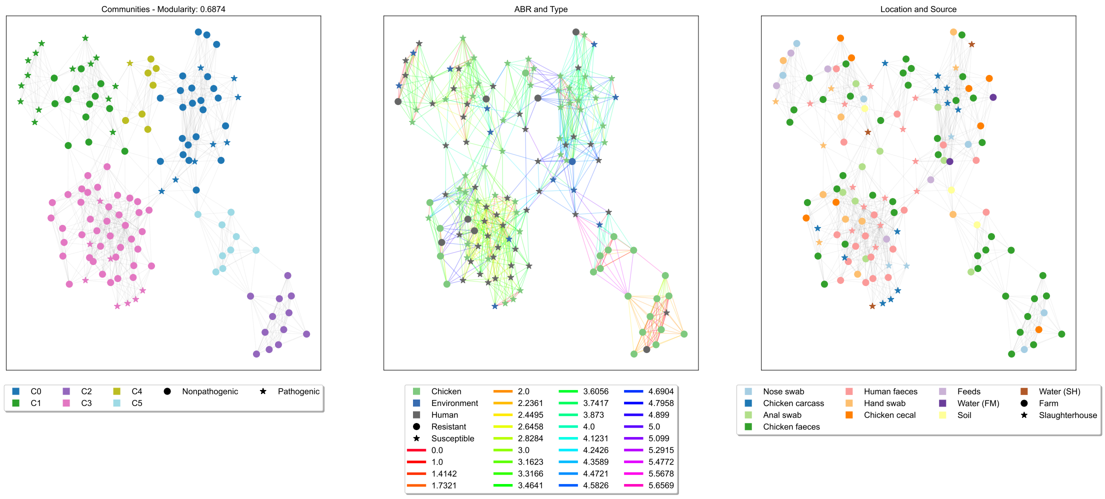

(ii)

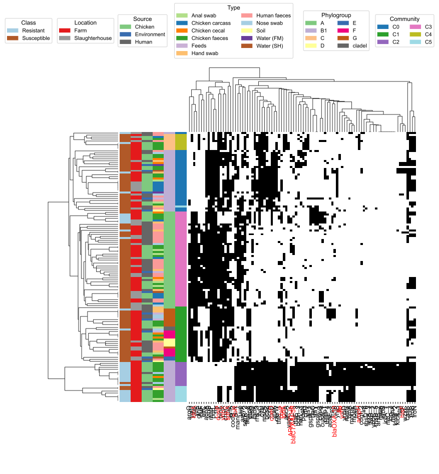

E. Cefoxitin (i)

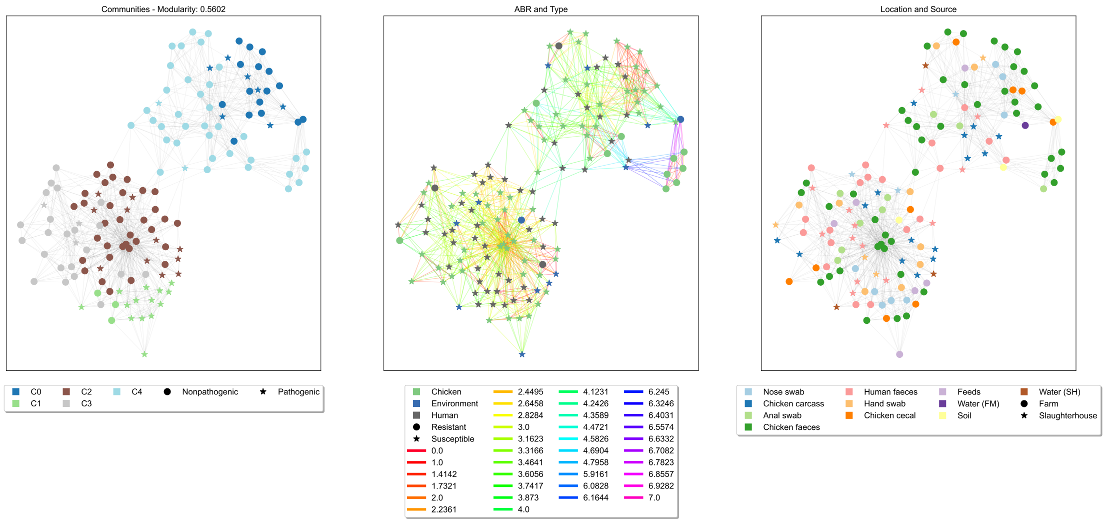

(ii)

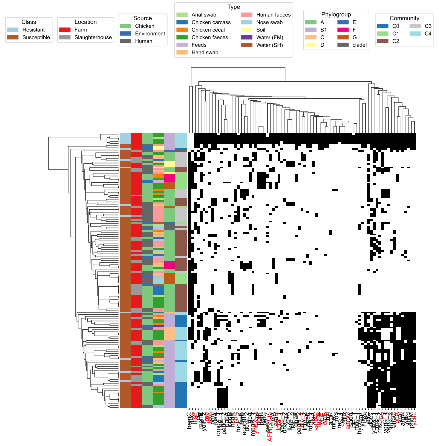

F. Chloramphenicol (i)

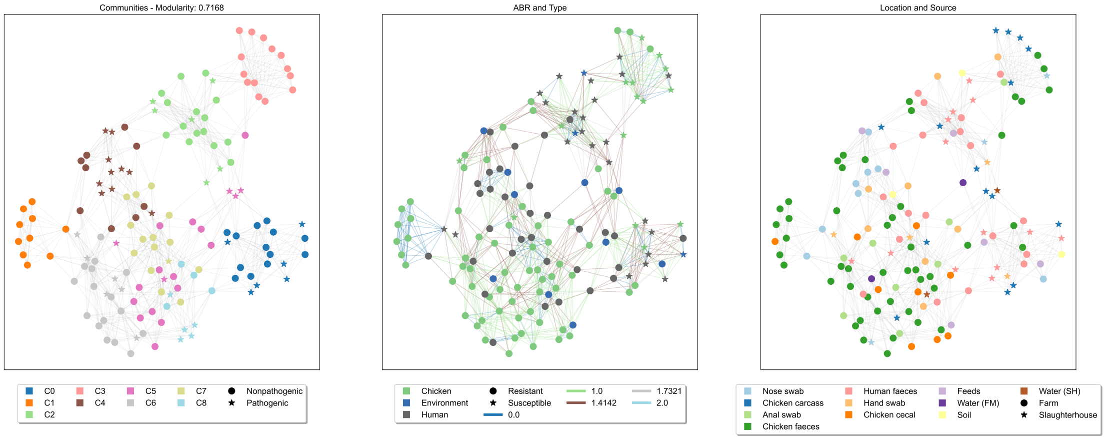

(ii)

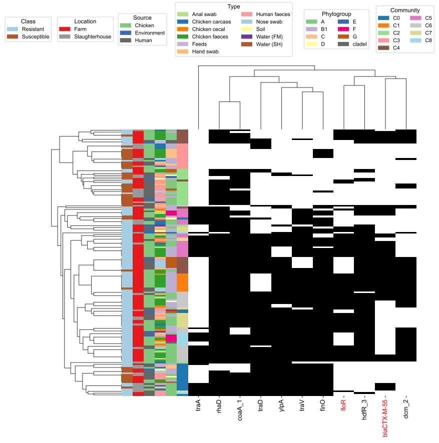

G. Ciprofloxacin (i)

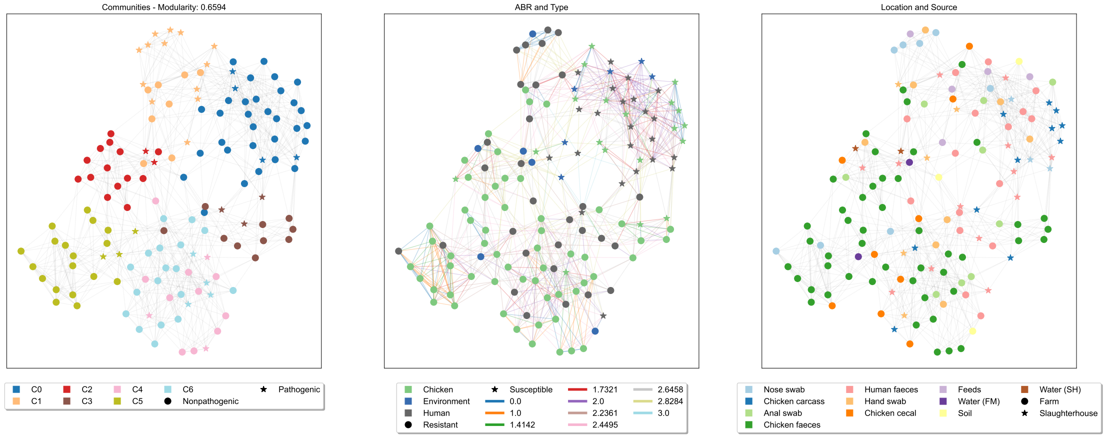

(ii)

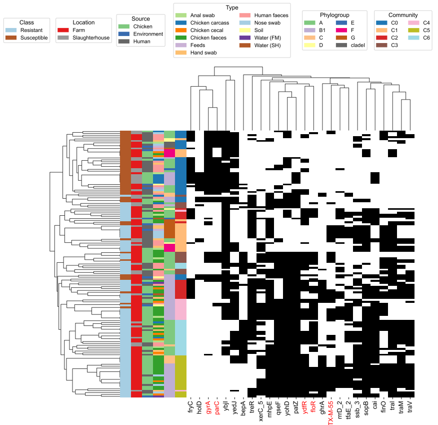

H. Cefotaxime (i)

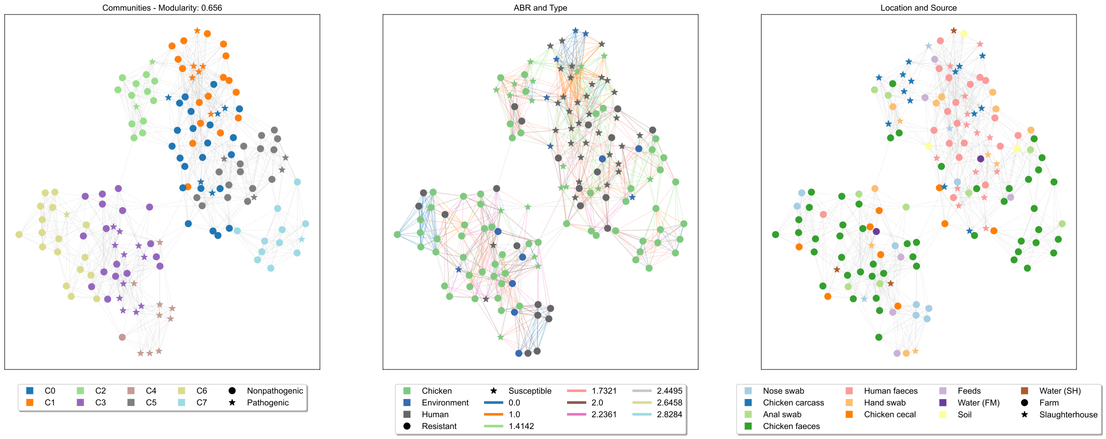

(ii)

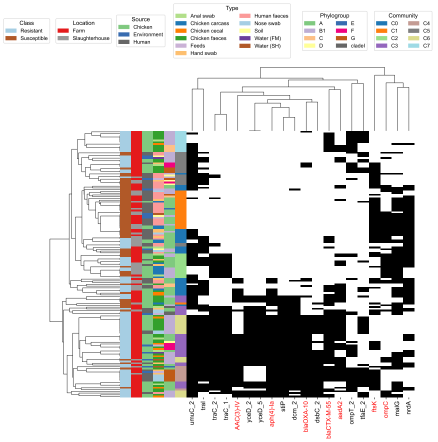

I. Doxyclyne (i)

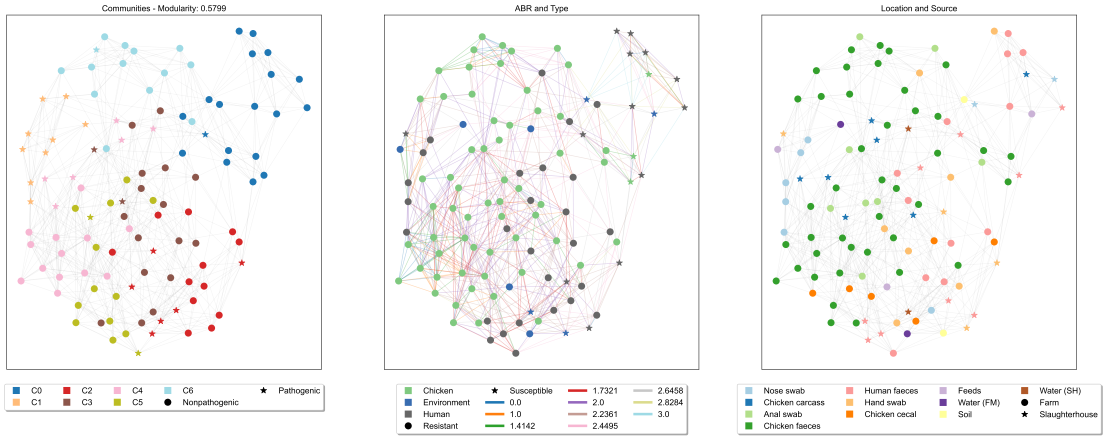

(ii)

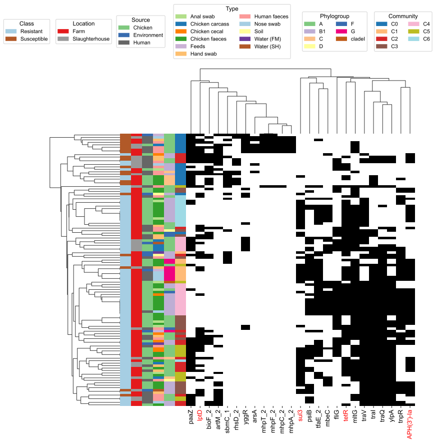

J. Cefalozin (i) (ii)

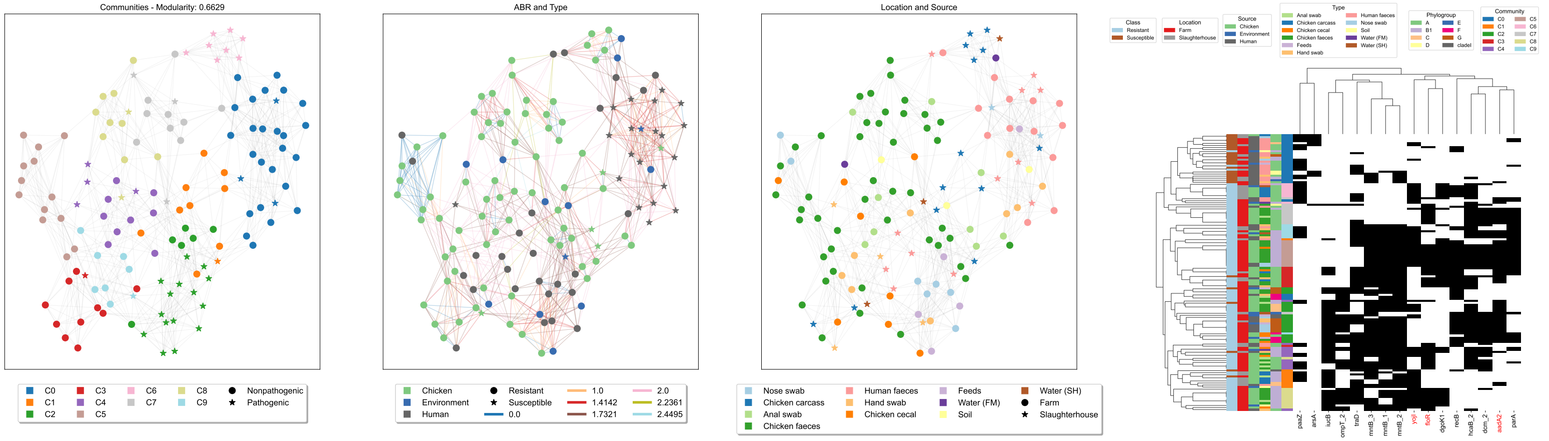

K. Gentamycin (i) (ii)

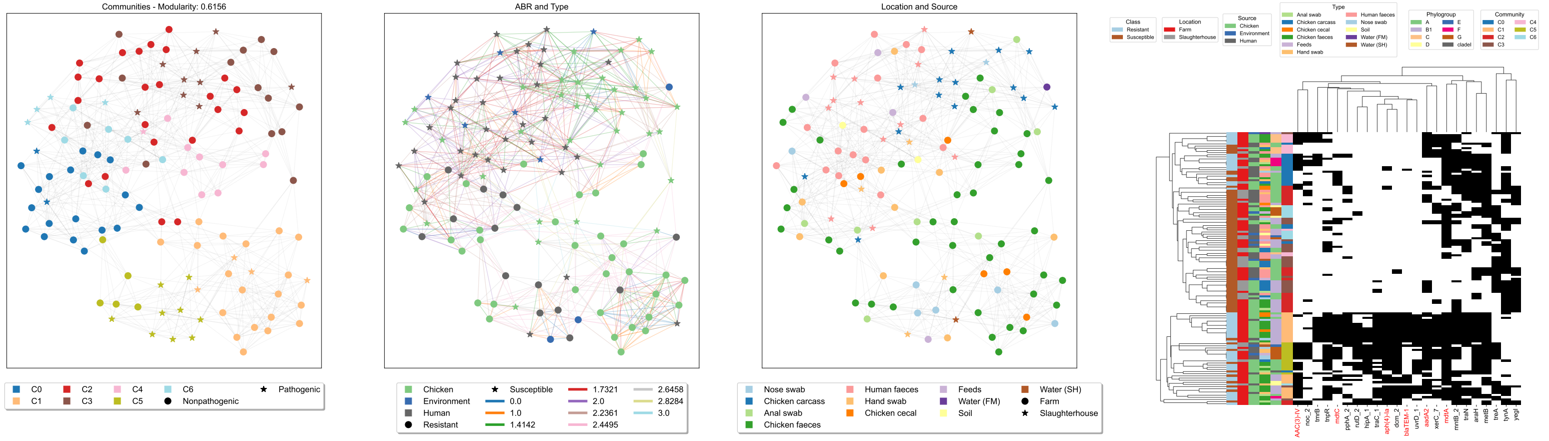

L. Levofloxacin (i) (ii)

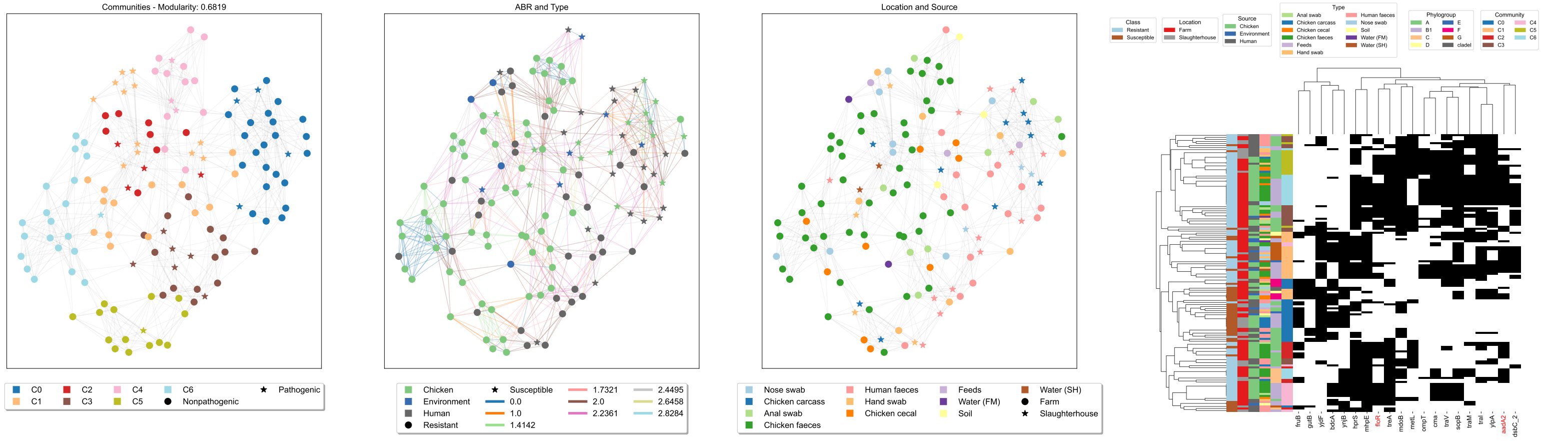

M. Minocycline (i)

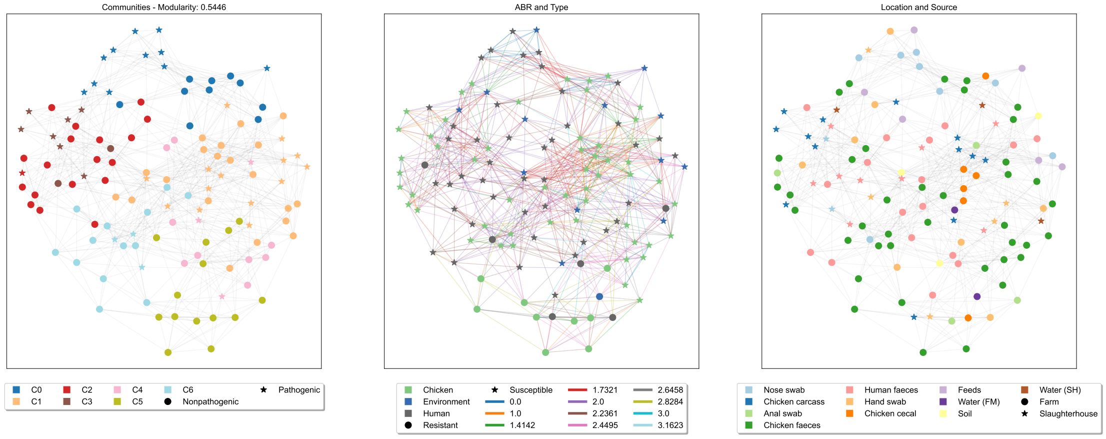

(ii)

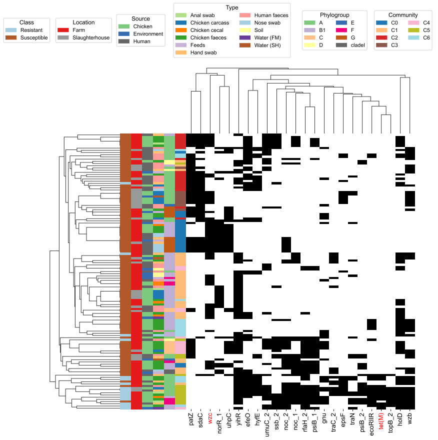

N. Nalidixic acid (i)

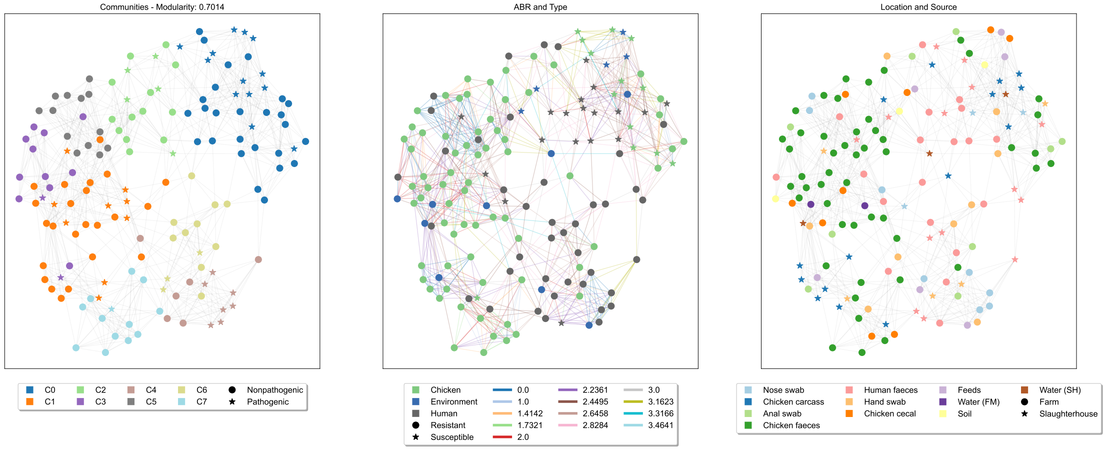

(ii)

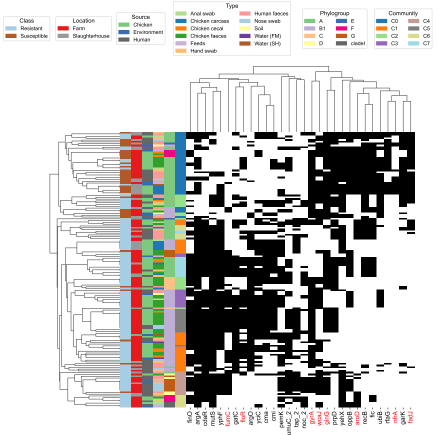

O. Streptomycin (i)

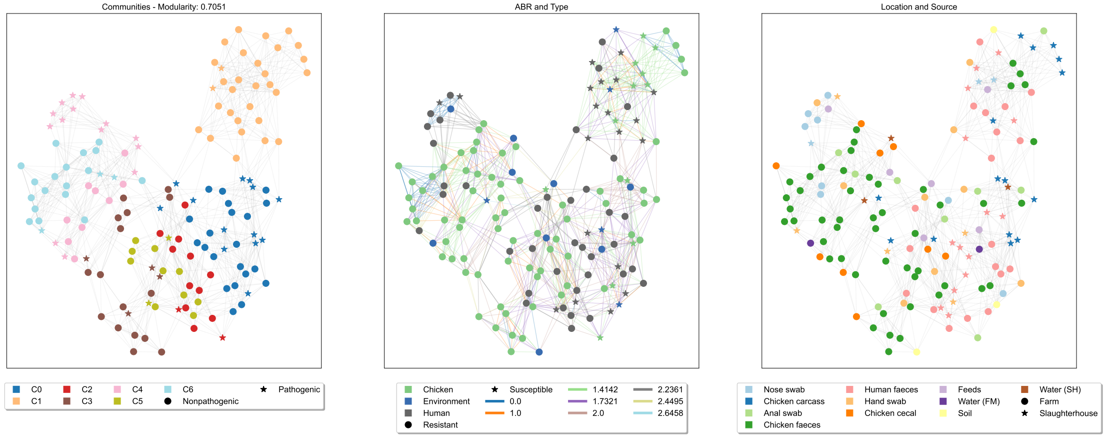

(ii)

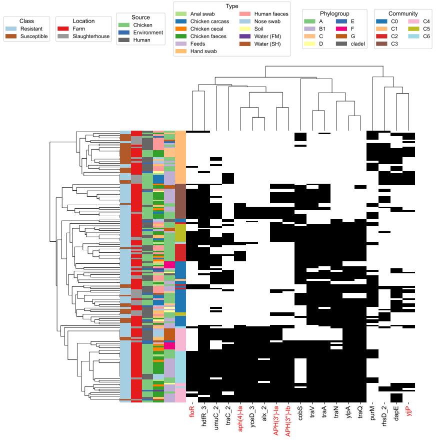

P. Sulfisoxazole (i)

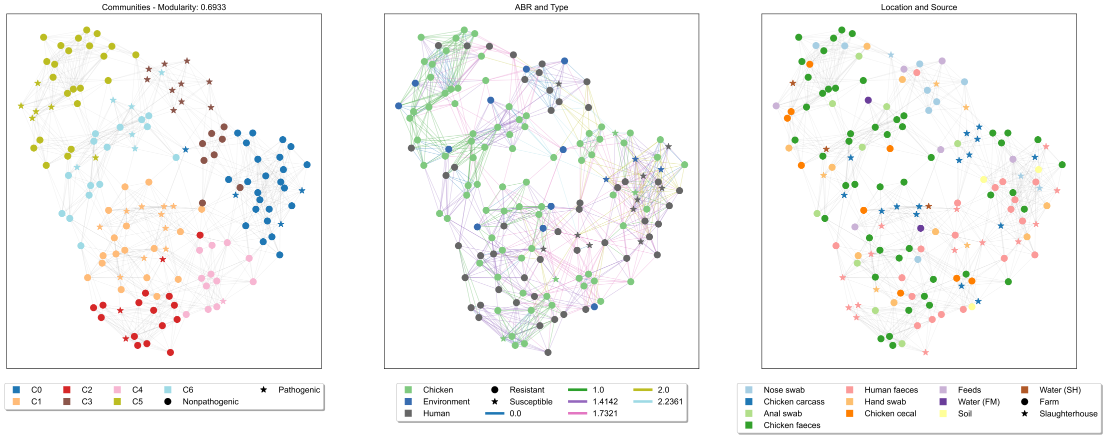

(ii)

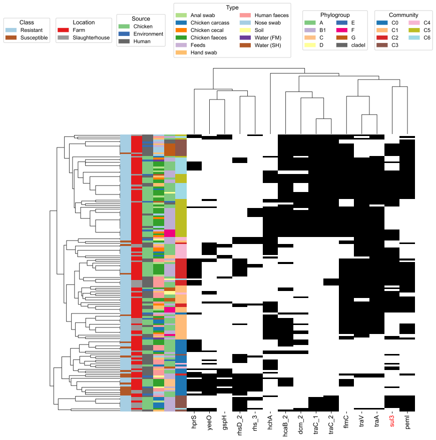

Q. Trimethroprim/Sulfamethoxazole (i)

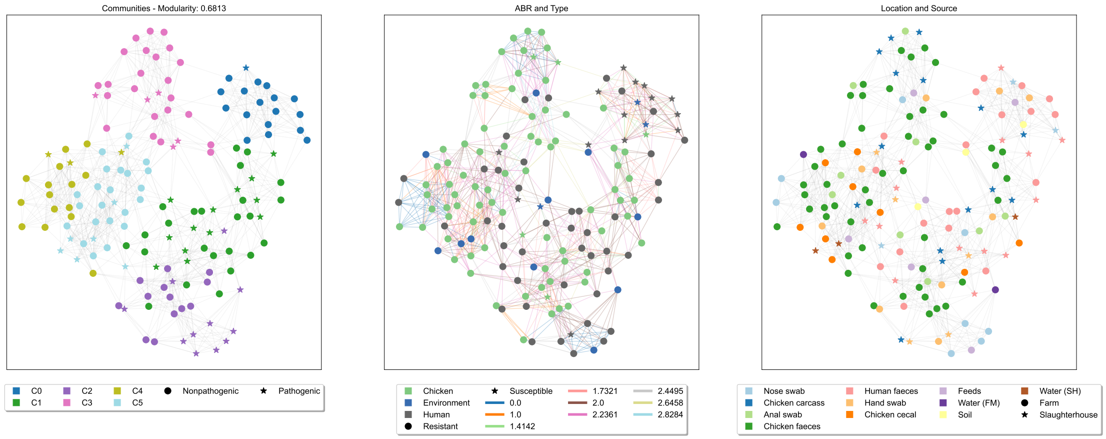

(ii)

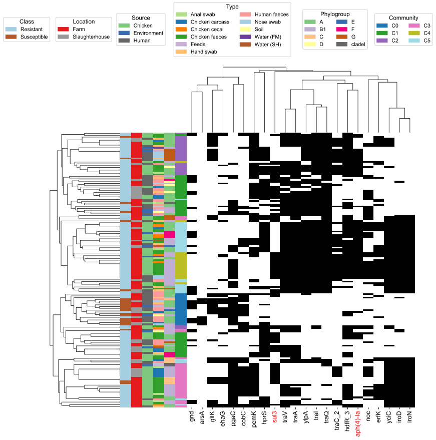

R. Tetracycline (i)

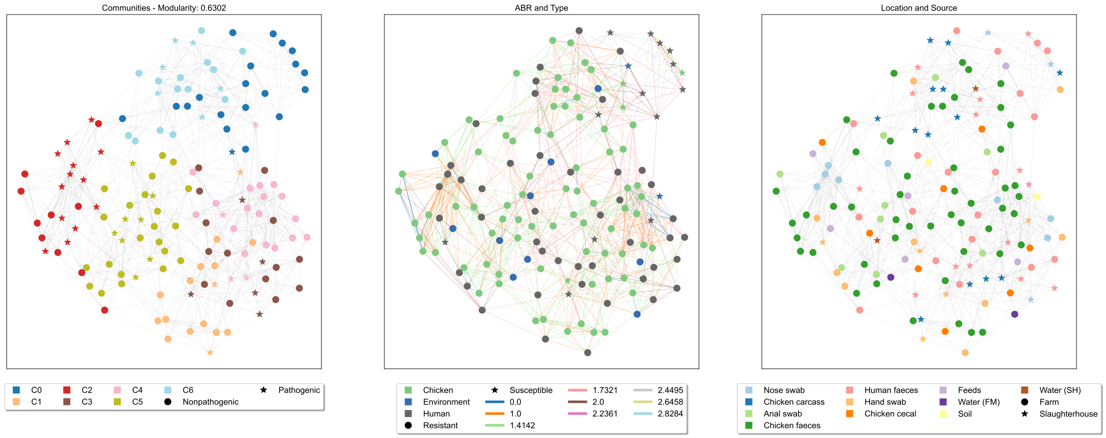

(ii)

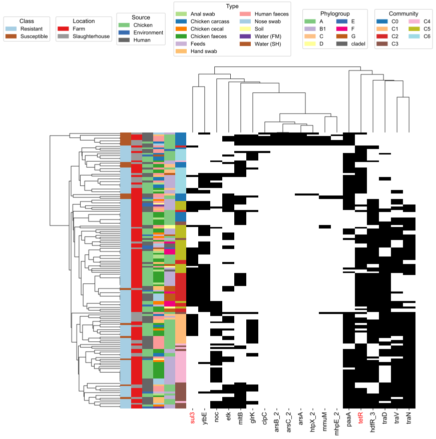

Supplement: S4 Fig — The resistome networks for amoxycillin/clavulanic acid, ampicillin, aztreonam, ceftazidime, cefoxitin, chloramphenicol, ciprofloxacin, cefotaxime, doxycycline, cefazolin, gentamycin, levofloxacin, minocycline, nalidixic acid, streptomycin, sulfisoxazole, trimethoprim/sulfamethoxazole and tetracycline from (A) to (R). For each network: (i) Panel on the left, indicates the communities and their respective numbers found using Louvain heuristics. Each community is indicated with a distinct colour. Each community is a set of nodes or clusters, that are densely and connected with statistical significance and identically coloured. For each network the node represents a sample and is shown in a distinct colour and shape depending on the metadata of the sample (e.g., source, AMR profile, location). In all panels the nodes are separated according to the Euclidean distance between the isolates; The central panel shows the AMR phenotype (resistant or susceptible) as the shape of the node and the source of the samples (human, chicken or environmental) as the colour of the node. An edge represents the Euclidean distance between two samples, and it is coloured according to the associated statistical value. The panel on the right indicates the location of the sample (farm or slaughterhouse) as the shape of the node and the type of sample as the colour of the node. (ii) Clustermap showing the genes associated with the AR discriminant k-mers used to build the networks. The columns on the left show the metadata (Class, Location, Source, Type, Clonal Complex, Phylogroup, Community number). The presence of the genes (in terms of its related k-mers) is indicated in black, while the absence is indicated in white). (PDF) [file pcbi.1010018.s004.pdf]
