# Supplementary material for: Whole-genome sequencing and gene sharing network analysis powered by machine learning identifies antibiotic resistance sharing between animals, humans and environment in livestock farming
Source: PLoS Comput Biol. 2022 Mar 25;18(3):e1010018. doi: 10.1371/journal.pcbi.1010018 (PMC8986120; doi:10.1371/journal.pcbi.1010018)
Supplement: Attachment — Submitted filename: ResponsetoReviewers.docx [file pcbi.1010018.s014.docx]

**Response to Reviewers**

We thank the Editor and Reviewers for their useful comments, which no doubt contributed to make this a better paper. In the following, a point-by-point response to all the questions and comments is provided. The original questions are in blue, our replies in black.

Editor’s comments

Thank you very much for submitting your manuscript "Whole genome sequencing and gene sharing network analysis powered by machine learning identifies antibiotic resistance sharing between animals, humans and environment in livestock farming" for consideration at PLOS Computational Biology.

As with all papers reviewed by the journal, your manuscript was reviewed by members of the editorial board and by several independent reviewers. In light of the reviews (below this email), we would like to invite the resubmission of a significantly-revised version that takes into account the reviewers' comments.

The reviewers have raised significant concerns about the method and scope, which need to be addressed before the manuscript can be considered for publication.

Primary concerns expressed were that:

- The novelty and choice of the methods have not been adequately justified.

- The machine learning pipeline needs further details.

- The study needs a better contextualisation to highlight the novelty.

- The sample size is fairly small and therefore the findings may not be generalisable to other settings. The small size may also affect the performance of the models.

- The raw data and annotated assemblies need to be shared in public repositories, with accession numbers provided in the manuscript.

We are pleased to be offered the chance to provide a revised manuscript for your consideration.

Items included in this submission:

i. Response to reviewers.
ii. Revised manuscript marked-up copy (Manuscript_markedup.docx)
iii. Revised manuscript clean copy (Manucript.docx)
iv. Supplementary Figures 1-5 and Supplementary Tables 1,3-6 – unchanged from previous version.
v. New Supplementary Table 2 generated to answer to question 8 of Reviewer 1.
vi. Revised Figure 2 to answer to questions 2 and 7 of Reviewer 1
vii. Figures 1, 3-6 – edited to the correct format as required for revised submission and as detailed in the decision letter.
viii. Original Supplementary Figures 2, 3, 4 and 5 had their names updated to Supplementary Figures 4, 5, 6 and 7, respectively, due to the addition of new Supplementary Figures 2 and 3.
ix. New Supplementary Figure 2 was generated to answer to question 8 of Reviewer 1 and question 3 of Reviewer 2.
x. New Supplementary Figure 3 was generated to answer to questions 1 and 3 of Reviewer 2.

**Reviewer #1**

In this study, the authors proposed a machine learning model to identify antibiotic resistance sharing between animals, humans and environment in livestock farming. Whole genome sequencing and gene sharing network analysis are used. The authors reached a promising performance, however, some major points should be addressed

We are delighted and thanks the reviewer for recognising the promising performance of our machine learning pipeline.

1. More literature review should be added to show more related works to this study.

We thank the reviewer for this suggestion and acknowledge that more literature review would help contextualise our results for readers. To this aim we have added additional review to the Introduction on lines 109-114 and Discussion on lines 652-659 and 681-762 and as below:

Introduction lines 109-114: “Several recent studies have undertaken to characterise the genome of *E. coli* isolates in relation to AMR in poultry farms (Nguyen VT *et al.* 2019; Aworh *et al.* 2021; Leekitcharoenphon *et al.* 2021). In addition, several papers have used machine learning to predict AMR phenotypes from both *E. coli* isolates (Her and Wu 2018; Moradigaravand *et al.* 2018; Aytan-Aktug *et al.* 2020; Hyun *et al*. 2020; Pearcy *et al.* 2021; Ren *et al.* 2021; Sunuwar and Azad 2021) and other bacteria (Kavvas *et al*. 2018, Liu *et al*. 2020, ValizadehAslani *et al.* 2020, Wang *et al.* 2021). To the best of our knowledge this is the first One Health comprehensive analysis and prediction of *E. coli* genome features related to AMR phenotypes, within an intensive poultry farm and connected slaughterhouse.”

Discussion lines 652-659: “Our isolates showed similar phylogroups and AMR profiles to a recent very similar cohort (human, broiler and environment isolates taken from poultry farm and market) sampled in Nigeria (Aworh *et al.* 2021). Whilst some differences were observed in ST types, with the predominant ST type from our study, ST205, absent in the Nigerian study, there was a large overlap in ST types with 10 of the 14 ST types identified in the Nigerian study also present in ours. As with our study a wide range or resistance genes were observed including colistin resistance gene, *mcr*-1, however, a smaller range of fluroquinolone resistance genes were found in our study compared to the Nigerian one (2 compared to 10 in Nigeria).”

Discussion lines 681-762: “A recent study by Ren (Ren *et al.* 2021) also looked at prediction of *E. coli* resistance from human and animal isolates from two datasets, one they term the Giessen data, 987 whole genome *E. coli* sequences they collected as part of their study and a public dataset of 1509 *E. coli* strains taken from Moradigaravand et al. (2018). Ren tested both of these datasets against four antibiotics (ciprofloxacin, cefotaxime, ceftazidime and gentamicin) all of which we also tested allowing comparison between our study and theirs. There were several key differences between our study and theirs: the Giessen data in Ren are described as human and animal clinical samples with no source country given and the public data were retrieved from both human clinical and environmental collections in the UK, Pakistan, Syria, Sweden, the USA, and Belgium. In contrast our data were from interconnected human, animal and environmental sources on a single Chinese poultry farm and connected slaughterhouse. Ren used SNPs (called against *E. coli* K-12 reference) as input to their classifiers, with no feature selection, whereas we used k-mers and performed a two-step feature selection to reduce sample-feature ratio. Ren then used Logistic regression, linear SVM, Random Forest and convolutional neural network classifiers to predict AMR phenotypes. In our study we used 10 classifiers including 3 that Ren used (LR, SVM, RF). Comparing the four antibiotics models across the two studies, our machine learning models achieved equal or higher AUC and precision values compared to all four antibiotic models. The sensitivity of our models was the same or better compared to both the Giessen and public datasets for all antibiotics except ceftazidime, where our sensitivity was 0.81 compared to 0.74-0.90 in the Ren study. For each of their datasets, Ren took the 10 highest-ranking features from each antibiotic and associated them with the genes in the genome to identify the cognate markers of AMR. Excluding known AMR genes, they found 19 genes of interest. Interestingly, 14 out of these 19 genes (*nha*A, *rlm*C, *fli*I, *pep*B, *prl*C, *sod*A, *mur*B, *rlu*F, *yjf*F, *tre*R, *arg*I, *val*S, *fhu*F and *nad*R) were also selected by our machine learning algorithms, and 4 (*sod*A, *rlu*F, *tre*R and *arg*I) were found to be in the 361 most discriminating genes. This overlapping of genes despite the different sample sources and countries (predominantly clinical isolates from humans and animals of international origin, compared to isolates from healthy animals and humans from China in our study) gives further evidence of the robustness of the methods we have used in this study.

Despite the limited number of samples available in our study, when we compared our machine learning performance metrics to published studies with similar and larger *E. coli* datasets we found comparable AUC, sensitivity, and specificity values (Her and Wu 2018, Moradigaravand *et al.* 2018, Hyun *et al.* 2020, Ren *et al.* 2021), indicating that the methods employed in this study have not been excessively affected by this limitation. Specifically, Hyun (Hyun *et al.* 2020) used 1588 *E. coli* genomes from publicly available data to build SVM-based prediction models using amino acid sequence variants. The genomes used were of human origin and predominantly from the UK and USA. Hyun tested six antibiotics of which four overlapped with those in our study (AMC, CAZ, CIP and GEN). Compared to Hyun across these 4 antibiotics we achieved better AUC values in our study for 3 of 4 models (AMC: 0.83 vs 0.91, CAZ: 0.95 vs 0.97, GEN: 0.94 vs 0.95 [Hyun vs ours]), with our AUC for CIP slightly worse with a value of 0.97 compared to 0.98. Our sensitivity (recall) was better than in Hyun for all 4 antibiotics. Our models performed slightly worse against Hyun in three antibiotics for accuracy (CAZ: 0.97 vs 0.92, CIP: 0.98 vs 0.94, GEN: 0.97 vs 0.90 [Hyun vs ours]) and precision (CAZ: 0.87 vs 0.88, CIP: 0.94 vs 0.96, GEN: 0.86 vs 0.94 [Hyun vs ours]). However, our AMC model performed better for both precision (0.67 vs 0.80) and accuracy (0.79 vs 0.85).

Similarly, in Moradigaravand (Moradigaravand *et* *al.* 2018) 1936 *E. coli* sequences (of which 1509 were also used in Ren, 2018) from human clinical and environmental sources were retrieved from collections in the UK, Pakistan, Syria, Sweden, the USA, and Belgium and used to predict AMR phenotypes using accessory gene presence-absence, matrix of the population structure, core genome SNPs, matrix of indels (insertions and deletions) and the year of isolation as features. Four machine learning classifiers were considered Random Forest, Gradient boosted decision trees, deep neural networks and logistic regression. Eleven antibiotics models were considered in Moradigaravand of which 6 overlapped with those of our study: AMC, AMP, CIP, CTX, CAZ (denoted CTZ in Moradigaravand) and GEN. Of these 6 antibiotics our dataset had better accuracy in 3 of 6 models (AMC: 0.81 vs 0.88, AMP: 0.93 vs 0.98, CIP 0.93 vs 0.94, CTX 0.97 vs 0.89, CAZ: 0.95 vs 0.93, and GEN: 0.97 vs 0.90 [Moradigaravand vs ours]). Our study had the same or better sensitivity (denoted R.RCL in (Moradigaravand *et al.* 2018)) in 5 of the 6 models (AMC: 0.64 vs 0.89, AMP: 0.96 vs 0.98, CIP 0.81 vs 0.97, CTX 0.92 vs 0.91, CAZ: 0.80 vs 0.80, and GEN: 0.81 vs 0.91 [Moradigaravand vs ours]). Comparing specificity our study achieved same or better specificity (denoted S.RCL in (Moradigaravand *et al*. 2018)) in 3 of 6 models (AMC: 0.60 vs 0.88, AMP: 0.96 vs 0.94, CIP 0.87 vs 0.88, CTX 0.93 vs 0.87, CAZ: 0.92 vs 0.96, and GEN: 0.98 vs 0.89 [Moradigaravand vs ours]).

Another *E coli* study with a smaller sample size compared to ours was that of Her and Wu (Her and Wu 2018). Her took 59 public database sequences (51 USA human isolates, 1 Taiwan human isolate, 1 Brazilian penguin isolate and 6 of unknown origin) and built machine learning predictors using radial SVM, Naïve Bayes, Adaboost, and Random Forest classifiers. Four different feature sets were used: core + accessory gene clusters, accessory gene clusters, CARD gene clusters and accessory gene cluster with CARD annotation. Of these the core + accessory gene cluster feature set is the most comparable with our k-mer features that cover both the core and accessory genome. Her and Wu studied 12 antibiotics of which eight overlapped with those studied in our work (AMP, AMS, CFZ, CAZ, FEP, GEN, CIP and SXT). Compared to Her and Wu (Her and Wu 2018) (looking at the most comparable feature set core + accessory gene clusters) our model gave the same or better AUC values for all the classifiers used in Her (AMP: 0.75 vs 1.00, AMS: 0.70 vs 0.97, CAZ: 0.84 vs 0.97, CFZ: 0.82 vs 1.00, FEP: 0.85 vs 0.94, GEN: 0.72 vs 0.95, CIP: 0.78 vs 0.97 and SXT 0.66 vs 0.99 [Her vs Ours]). Additionally, we achieved equal or better sensitivity values across 5 of 8 antibiotics compared Her and Wu (AMP: 0.5 vs 0.98, AMS: 0.53 vs 0.99, CAZ: 0.80 vs 0.80, CFZ: 0.86 vs 0.98, FEP: 0.90 vs 0.89, GEN: 1.00 vs 0.91, CIP: 1.00 vs 0.97 and SXT 0.49 vs 0.97 [Her vs Ours]), and better precision also in 5 antibiotics (AMP: 1.00 vs 0.99, AMS: 1.00 vs 0.95, CAZ: 0.81 vs 0.87, CFZ: 0.79 vs 0.99, FEP: 0.82 vs 0.89, GEN: 0.85 vs 0.86, CIP: 0.73 vs 0.94 and SXT 1.00 vs 0.98 [Her vs Ours]).”

Aworh MK, Kwaga JKP, Hendriksen RS, Okolocha EC, Thakur S. Genetic relatedness of multidrug resistant Escherichia coli isolated from humans, chickens and poultry environments. Antimicrobial Resistance & Infection Control. 2021;10(1):58.

Aytan-Aktug D, Clausen PTLC, Bortolaia V, Aarestrup FM, Lund O, Marshall CW. Prediction of Acquired Antimicrobial Resistance for Multiple Bacterial Species Using Neural Networks. mSystems. 2020;5(1):e00774-19.

Her H-L, Wu Y-W. A pan-genome-based machine learning approach for predicting antimicrobial resistance activities of the Escherichia coli strains. Bioinformatics. 2018;34(13):i89-i95.

Hyun JC, Kavvas ES, Monk JM, Palsson BO. Machine learning with random subspace ensembles identifies antimicrobial resistance determinants from pan-genomes of three pathogens. PLOS Computational Biology. 2020;16(3):e1007608.

Kavvas ES, Catoiu E, Mih N, Yurkovich JT, Seif Y, Dillon N, et al. Machine learning and structural analysis of Mycobacterium tuberculosis pan-genome identifies genetic signatures of antibiotic resistance. Nature Communications. 2018;9(1):4306.

Leekitcharoenphon P, Johansson MHK, Munk P, Malorny B, Skarżyńska M, Wadepohl K, et al. Genomic evolution of antimicrobial resistance in Escherichia coli. Scientific Reports. 2021;11(1):15108.

Liu Z, Deng D, Lu H, Sun J, Lv L, Li S, et al. Evaluation of Machine Learning Models for Predicting Antimicrobial Resistance of Actinobacillus pleuropneumoniae From Whole Genome Sequences. Frontiers in Microbiology. 2020;11.

Moradigaravand D, Palm M, Farewell A, Mustonen V, Warringer J, Parts L. Prediction of antibiotic resistance in Escherichia coli from large-scale pan-genome data. Plos Computational Biology. 2018;14(12):e1006258.

Nguyen VT, Jamrozy D, Matamoros S, Carrique-Mas JJ, Ho HM, Thai QH, et al. Limited contribution of non-intensive chicken farming to ESBL-producing Escherichia coli colonization in humans in Vietnam: an epidemiological and genomic analysis. Journal of Antimicrobial Chemotherapy. 2019;74(3):561-70.

Pearcy N, Hu Y, Baker M, Maciel-Guerra A, Xue N, Wang W, et al. Genome-Scale Metabolic Models and Machine Learning Reveal Genetic Determinants of Antibiotic Resistance in Escherichia coli and Unravel the Underlying Metabolic Adaptation Mechanisms. mSystems. 2021;6(4):e00913-20.

Ren Y, Chakraborty T, Doijad S, Falgenhauer L, Falgenhauer J, Goesmann A, et al. Prediction of antimicrobial resistance based on whole-genome sequencing and machine learning. Bioinformatics (Oxford, England). 2021;38(2):325-34.

Sunuwar J, Azad RK. A machine learning framework to predict antibiotic resistance traits and yet unknown genes underlying resistance to specific antibiotics in bacterial strains. Briefings in Bioinformatics. 2021;22(6).

ValizadehAslani T, Zhao Z, Sokhansanj BA, Rosen GL. Amino Acid k-mer Feature Extraction for Quantitative Antimicrobial Resistance (AMR) Prediction by Machine Learning and Model Interpretation for Biological Insights. Biology. 2020;9(11):365.

Wang W, Baker M, Hu Y, Xu J, Yang D, Maciel-Guerra A, et al. Whole-Genome Sequencing and Machine Learning Analysis of Staphylococcus aureus from Multiple Heterogeneous Sources in China Reveals Common Genetic Traits of Antimicrobial Resistance. mSystems. 2021;6(3):e01185-20.

2. The authors used logistic regression, linear support vector machine and radial basis function kernel support vector machine (RBF-SVM) as their algorithms. Why was extra tree used in Fig. 2?

We apologise for any confusion regarding this. The pipeline for the machine learning has three stages: pre-processing, ML prediction and post-processing. The extra tree classifier is used only in pre-processing as part of the feature selection and is not used as a prediction classifier. The extra tree classifier was used to acquire the Gini feature importance of each feature. The features with an overall mean importance higher than the average mean are used as an input to train and test the following classifiers: Logistic Regression, Linear SVM, RBF-SVM, Extra Tree Classifier, Random Forest, Adaboost, XGBoost, Naïve Bayes, Linear Discriminant Analysis and Quadratic Discriminant Analysis. Whilst this was explained in the methods, we acknowledge that it was not clear when reading the results and so have now added extra clarification in the Results on lines 259-263 and below:

Results lines 259-263: “To reduce the number of features analysed by the classifiers a two-step feature selection method was applied: firstly, k-mers with a p-value greater than 0.05 according to the chi-square test were discarded (Aun et al 2018, Nguyen et al. 2019) and then those remaining were used as input to an Extra Tree Classifier. K-mers with a Gini feature importance above the overall mean were selected (Song et al. 2020, Allen et al. 2021).”

Additionally, we have edited the pipeline in Figure 2A to add clarity to which steps are part of the pre-processing, which are part of the prediction methods, and which are the post-processing steps. See the new Figure 2 for the manuscript below:

Figure 2 Legend lines 284-299: “Fig 2. Supervised machine learning prediction of antimicrobial resistance signature profiles to 21 antimicrobials in the E. coli cohort. A) Flow diagram showing the machine learning pipeline including feature selection (orange), classification (blue), post-processing (green); B) Prediction performance results of the RBF SVM classifier that achieved the best performance amongst the three investigated is shown. Five performance indicators have been used to evaluate the classification: i) Area under the curve AUC, ii) Accuracy, iii) Sensitivity, iv) Precision, v) Specificity, and vi) Cohen’s Kappa value from 30 training runs for each antimicrobial. The scores for each performance metric are indicated in the Y axis. Predictive models were generated to classify the resistance vs. susceptibility profiles of twenty-one different antimicrobials (X axis): amoxycillin/clavulanic acid (AMC), ampicillin (AMP), ampicillin/sulbactam (AMS), aztreonam (AZM), ceftazidime (CAZ), cefoxitin (CFX), cefazolin (CFZ), chloramphenicol (CHL), ciprofloxacin (CIP), cefotaxime (CTX), doxycycline (DOX), cefepime (FEP), gentamicin (GEN), kanamycin (KAN), levofloxacin (LEV), minocycline (MIN), nalidixic acid (NAL), streptomycin (STR), trimethoprim/sulfamethoxazole (SXT), sulfisoxazole (SUL) and tetracycline (TET).”

Allen JP, Snitkin E, Pincus NB, Hauser AR. (2021) Forest and Trees: Exploring Bacterial Virulence with Genome-wide Association Studies and Machine Learning. *Trends Microbiol.*;29(7):621-633.

Aun E, Brauer A, Kisand V, Tenson T, Remm M. (2018) A k-mer-based method for the identification of phenotype-associated genomic biomarkers and predicting phenotypes of sequenced bacteria. *PLoS Comput Biol*. 2018;14(10):e1006434.

Nguyen M, Long SW, McDermott PF, Olsen RJ, Olson R, Stevens RL, Tyson GH, Zhao S, Davis JJ. (2019) Using Machine Learning To Predict Antimicrobial MICs and Associated Genomic Features for Nontyphoidal Salmonella. *J Clin Microbiol*. 57(2):e01260-18

Song K, Wright FA, Zhou YH. (2020) Systematic Comparisons for Composition Profiles, Taxonomic Levels, and Machine Learning Methods for Microbiome-Based Disease Prediction. *Front Mol Biosci* 16;7:610845

3. The use of inconsistent cross-validation method, i.e., sometimes using nested CV, sometimes using 5-fold or 3-fold.

We apologise for any confusion regarding the cross-validation method. In this work we chose a nested cross validation approach where a 3-fold cross-validation procedure for model hyper-parameter optimization (inner loop) is nested inside the 5-fold cross-validation procedure for model selection (outer loop). The nested cross-validation method is an approach used for model hyper-parameter optimization and model selection that attempts to overcome overfitting issues during the training phase. The use of two cross-validation loops also leads the procedure to be called “double cross-validation” formed by two connected cross validations. The diagram below describes how a nested cross validation works (Kumar 2020). The original set of data is first split into a training and testing folds on the outer loop, after the training set is further split into training and validation sets in the inner loop. Therefore, the inner loop is used to maximise the model score by fitting a model on the training set and testing the model with optimal combination of hyper parameters on the validation set. Once the most optimal model is selected using the inner loop, the generalization error of the model is estimated using the cross-validation technique in the outer loop. In our work a 3-fold cross-validation procedure for model hyper-parameter optimization (inner loop) is nested inside the 5-fold cross-validation procedure for model selection (outer loop). The number of folds in the outer and inner loop were defined following the work done by Wainer & Cawley (2017). According to Vabalas *et al.,* 2019 the nested cross validation approach can give an unbiased performance estimate regardless of the sample size, they indicated that by using a nested cross validation approach the models were able to learn patterns in the data more efficiently and achieved high performance results. They concluded that completely separating testing and training data is enough to obtain unbiased performance estimates regardless of sample size.

Nested cross validation with a 3-fold inner loop and a 5-fold outer loop (Kumar 2020)

We acknowledge that we did not describe these methods fully in the manuscript. We have now updated the methods to add better explain the methodology, so it is now clearer, see lines 1079-1088 and below:

Method lines 1079-1088: “Nested Cross-validation (NCV) (Cawley & Talbot, 2010) was employed to assess the performance and select the hyper-parameters of the proposed classifiers. In NCV, an outer loop (5-fold) split the data set into test and training sets. For each training set, a grid search (inner loop, 3-fold) was run, to find the best hyper-parameters of the classifier using accuracy as a performance metric. Then, the test set was used to score the best classifier found in the inner loop. Thirty iterations of each classifier were carried out, wherein each iteration an NCV was employed. The inner loop of the NCV found the best hyper-parameters of each classifier using stratified 3-fold cross-validation; the outer loop measured the ROC-AUC (receiver operating characteristic area under the curve) accuracy, sensitivity, specificity, precision and Cohen’s kappa of the test data set (unseen in the inner loop for the training) using 5-fold stratified cross-validation, to compare all the classifiers (Wainer & Cawley, 2017).”

Cawley GC, Talbot NLC. On over-fitting in model selection and subsequent selection bias in performance evaluation. Journal of Machine Learning Research. 2010;11:2079-107

Kumar A. Python – Nested Cross Validation for Algorithm Selection 2020 [cited 2022 23/01/2022]. Nested Cross Validation]. Available from: <https://vitalflux.com/python-nested-cross-validation-algorithm-selection>.

Vabalas A, Gowen E, Poliakoff E, Casson AJ (2019) “Machine learning algorithm validation with a limited sample size”. PLOS ONE 14(11): e0224365.

Wainer J, Cawley G. Empirical evaluation of resampling procedures for optimising SVM hyperparameters. Journal of Machine Learning Research. 2017;18:475-509.

4. This study requires an external validation data to evaluate the performance of models on unseen data.

We thank the reviewer for its comment. As explained in the previous answer (answer to comment n.3) we have used an external validation set. Specifically, this work used a nested cross validation approach to select the model with a hyper-parameter optimization. The outer loop of this approach divides the data into a training and a test fold. The test fold is used for external validation, since the labels are kept unseen during the training of the model (inner loop), and the performance metrics are evaluated only in the test fold. The metrics are averaged across the 5-fold cross validation of the outer loop, and the final results are the mean of the 30 iterations done for the nested cross validation, i.e., the nested cross validation is run 30 times with different splits of the data set. However, to make the text clearer we have now updated the Method lines 1084-1088 to describe how the metrics are measured.

Method lines 1084-1088: “The inner loop of the NCV found the best hyper-parameters of each classifier using stratified 3-fold cross-validation; the outer loop measured the ROC-AUC (receiver operating characteristic area under the curve) accuracy, sensitivity, specificity, precision and Cohen’s kappa of the test data set (unseen in the inner loop for the training) using 5-fold stratified cross-validation, to compare all the classifiers (Wainer & Cawley, 2017).”

Wainer J, Cawley G. Empirical evaluation of resampling procedures for optimising SVM hyperparameters. Journal of Machine Learning Research. 2017;18:475-509.

5. The authors should compare the predictive performance to previously published works on the same problem/data.

We thank the reviewer for this suggestion and have added additional comparison to the discussion on lines 681-762 and below:

Discussion lines 681-762: “A recent study by Ren (Ren *et al.* 2021) also looked at prediction of *E. coli* resistance from human and animal isolates from two datasets, one they term the Giessen data, 987 whole genome *E. coli* sequences they collected as part of their study and a public dataset of 1509 *E. coli* strains taken from Moradigaravand et al. (2018). Ren tested both of these datasets against four antibiotics (ciprofloxacin, cefotaxime, ceftazidime and gentamicin) all of which we also tested allowing comparison between our study and theirs. There were several key differences between our study and theirs: the Giessen data in Ren are described as human and animal clinical samples with no source country given and the public data were retrieved from both human clinical and environmental collections in the UK, Pakistan, Syria, Sweden, the USA, and Belgium. In contrast our data were from interconnected human, animal and environmental sources on a single Chinese poultry farm and connected slaughterhouse. Ren used SNPs (called against *E. coli* K-12 reference) as input to their classifiers, with no feature selection, whereas we used k-mers and performed a two-step feature selection to reduce sample-feature ratio. Ren then used Logistic regression, linear SVM, Random Forest and convolutional neural network classifiers to predict AMR phenotypes. In our study we used 10 classifiers including 3 that Ren used (LR, SVM, RF). Comparing the four antibiotics models across the two studies, our machine learning models achieved equal or higher AUC and precision values compared to all four antibiotic models. The sensitivity of our models was the same or better compared to both the Giessen and public datasets for all antibiotics except ceftazidime, where our sensitivity was 0.81 compared to 0.74-0.90 in the Ren study. For each of their datasets, Ren took the 10 highest-ranking features from each antibiotic and associated them with the genes in the genome to identify the cognate markers of AMR. Excluding known AMR genes, they found 19 genes of interest. Interestingly, 14 out of these 19 genes (*nha*A, *rlm*C, *fli*I, *pep*B, *prl*C, *sod*A, *mur*B, *rlu*F, *yjf*F, *tre*R, *arg*I, *val*S, *fhu*F and *nad*R) were also selected by our machine learning algorithms, and 4 (*sod*A, *rlu*F, *tre*R and *arg*I) were found to be in the 361 most discriminating genes. This overlapping of genes despite the different sample sources and countries (predominantly clinical isolates from humans and animals of international origin, compared to isolates from healthy animals and humans from China in our study) gives further evidence of the robustness of the methods we have used in this study.

Despite the limited number of samples available in our study, when we compared our machine learning performance metrics to published studies with similar and larger *E. coli* datasets we found comparable AUC, sensitivity, and specificity values (Her and Wu 2018, Moradigaravand *et al.* 2018, Hyun *et al.* 2020, Ren *et al.* 2021), indicating that the methods employed in this study have not been excessively affected by this limitation. Specifically, Hyun (Hyun *et al.* 2020) used 1588 *E. coli* genomes from publicly available data to build SVM-based prediction models using amino acid sequence variants. The genomes used were of human origin and predominantly from the UK and USA. Hyun tested six antibiotics of which four overlapped with those in our study (AMC, CAZ, CIP and GEN). Compared to Hyun across these 4 antibiotics we achieved better AUC values in our study for 3 of 4 models (AMC: 0.83 vs 0.91, CAZ: 0.95 vs 0.97, GEN: 0.94 vs 0.95 [Hyun vs ours]), with our AUC for CIP slightly worse with a value of 0.97 compared to 0.98. Our sensitivity (recall) was better than in Hyun for all 4 antibiotics. Our models performed slightly worse against Hyun in three antibiotics for accuracy (CAZ: 0.97 vs 0.92, CIP: 0.98 vs 0.94, GEN: 0.97 vs 0.90 [Hyun vs ours]) and precision (CAZ: 0.87 vs 0.88, CIP: 0.94 vs 0.96, GEN: 0.86 vs 0.94 [Hyun vs ours]). However, our AMC model performed better for both precision (0.67 vs 0.80) and accuracy (0.79 vs 0.85).

Similarly, in Moradigaravand (Moradigaravand *et* *al.* 2018) 1936 *E. coli* sequences (of which 1509 were also used in Ren, 2018) from human clinical and environmental sources were retrieved from collections in the UK, Pakistan, Syria, Sweden, the USA, and Belgium and used to predict AMR phenotypes using accessory gene presence-absence, matrix of the population structure, core genome SNPs, matrix of indels (insertions and deletions) and the year of isolation as features. Four machine learning classifiers were considered Random Forest, Gradient boosted decision trees, deep neural networks and logistic regression. Eleven antibiotics models were considered in Moradigaravand of which 6 overlapped with those of our study: AMC, AMP, CIP, CTX, CAZ (denoted CTZ in Moradigaravand) and GEN. Of these 6 antibiotics our dataset had better accuracy in 3 of 6 models (AMC: 0.81 vs 0.88, AMP: 0.93 vs 0.98, CIP 0.93 vs 0.94, CTX 0.97 vs 0.89, CAZ: 0.95 vs 0.93, and GEN: 0.97 vs 0.90 [Moradigaravand vs ours]). Our study had the same or better sensitivity (denoted R.RCL in (Moradigaravand *et al.* 2018)) in 5 of the 6 models (AMC: 0.64 vs 0.89, AMP: 0.96 vs 0.98, CIP 0.81 vs 0.97, CTX 0.92 vs 0.91, CAZ: 0.80 vs 0.80, and GEN: 0.81 vs 0.91 [Moradigaravand vs ours]). Comparing specificity our study achieved same or better specificity (denoted S.RCL in (Moradigaravand *et al*. 2018)) in 3 of 6 models (AMC: 0.60 vs 0.88, AMP: 0.96 vs 0.94, CIP 0.87 vs 0.88, CTX 0.93 vs 0.87, CAZ: 0.92 vs 0.96, and GEN: 0.98 vs 0.89 [Moradigaravand vs ours]).

Another *E coli* study with a smaller sample size compared to ours was that of Her and Wu (Her and Wu 2018). Her took 59 public database sequences (51 USA human isolates, 1 Taiwan human isolate, 1 Brazilian penguin isolate and 6 of unknown origin) and built machine learning predictors using radial SVM, Naïve Bayes, Adaboost, and Random Forest classifiers. Four different feature sets were used: core + accessory gene clusters, accessory gene clusters, CARD gene clusters and accessory gene cluster with CARD annotation. Of these the core + accessory gene cluster feature set is the most comparable with our k-mer features that cover both the core and accessory genome. Her and Wu studied 12 antibiotics of which eight overlapped with those studied in our work (AMP, AMS, CFZ, CAZ, FEP, GEN, CIP and SXT). Compared to Her and Wu (Her and Wu 2018) (looking at the most comparable feature set core + accessory gene clusters) our model gave the same or better AUC values for all the classifiers used in Her (AMP: 0.75 vs 1.00, AMS: 0.70 vs 0.97, CAZ: 0.84 vs 0.97, CFZ: 0.82 vs 1.00, FEP: 0.85 vs 0.94, GEN: 0.72 vs 0.95, CIP: 0.78 vs 0.97 and SXT 0.66 vs 0.99 [Her vs Ours]). Additionally, we achieved equal or better sensitivity values across 5 of 8 antibiotics compared Her and Wu (AMP: 0.5 vs 0.98, AMS: 0.53 vs 0.99, CAZ: 0.80 vs 0.80, CFZ: 0.86 vs 0.98, FEP: 0.90 vs 0.89, GEN: 1.00 vs 0.91, CIP: 1.00 vs 0.97 and SXT 0.49 vs 0.97 [Her vs Ours]), and better precision also in 5 antibiotics (AMP: 1.00 vs 0.99, AMS: 1.00 vs 0.95, CAZ: 0.81 vs 0.87, CFZ: 0.79 vs 0.99, FEP: 0.82 vs 0.89, GEN: 0.85 vs 0.86, CIP: 0.73 vs 0.94 and SXT 1.00 vs 0.98 [Her vs Ours]).”

Her, H. L. and Wu Y. W. (2018). "A pan-genome-based machine learning approach for predicting antimicrobial resistance activities of the Escherichia coli strains." Bioinformatics 34(13): i89-i95.

Hyun JC, Kavvas ES, Monk JM, Palsson BO. Machine learning with random subspace ensembles identifies antimicrobial resistance determinants from pan-genomes of three pathogens. PLOS Computational Biology. 2020;16(3):e1007608.

Moradigaravand, D., et al. (2018). "Prediction of antibiotic resistance in Escherichia coli from large-scale pan-genome data." PloS Computational Biology 14(12): e1006258.

Ren, Y., et al. (2021). "Prediction of antimicrobial resistance based on whole-genome sequencing and machine learning." Bioinformatics (Oxford, England) 38(2): 325-334.

6. Measurement metrics (i.e., AUC, Accuracy, Sensitivity, Specificity, ...) have been used in previous bioinformatics studies such as PMID: 34915158, PMID: 34812044. Thus, the authors are suggested to refer to more works in this description to attract a broader readership.

We thank the reviewer for this suggestion. We have now included additional explanation of these metrics in the Methods on lines 1092-1094 and below:

Methods lines 1092-1094: “The prediction metrics accuracy (TP+TN/(P+N)), sensitivity (true positive rate: TP/P), specificity (true negative rate: TN/N), Area Under the ROC Curve (AUC) and Cohen’s Kappa and precision were used, as typically used to measure ML performance, see (Pearcy *et al.* 2021, Wang *et al.* 2021, Müller and Guido 2016, Le and Ho 2021, Tng *et al*. 2022).”

Le NQK, Ho Q-T. Deep transformers and convolutional neural network in identifying DNA N6-methyladenine sites in cross-species genomes. Methods. 2021.

Müller AC, Guido S. Introduction to Machine Learning with Python: A Guide for Data Scientists: O'Reilly Media, Incorporated; 2016.

Pearcy N, Hu Y, Baker M, Maciel-Guerra A, Xue N, Wang W, et al. Genome-Scale Metabolic Models and Machine Learning Reveal Genetic Determinants of Antibiotic Resistance in Escherichia coli and Unravel the Underlying Metabolic Adaptation Mechanisms. mSystems. 2021;6(4):e00913-20.

Tng SS, Le NQK, Yeh H-Y, Chua MCH. Improved Prediction Model of Protein Lysine Crotonylation Sites Using Bidirectional Recurrent Neural Networks. Journal of Proteome Research. 2022;21(1):265-73. doi: 10.1021/acs.jproteome.1c00848.

Wang W, Baker M, Hu Y, Xu J, Yang D, Maciel-Guerra A, et al. Whole-Genome Sequencing and Machine Learning Analysis of Staphylococcus aureus from Multiple Heterogeneous Sources in China Reveals Common Genetic Traits of Antimicrobial Resistance. mSystems. 2021;6(3):e01185-20.

7. "..." parts in Fig. 2 should be filled completely.

We apologise for this mistake. The Figure 2 is now corrected with “…” remove and the complete information added. We have also included now a colour scheme to highlight the main parts of the pipeline (feature selection, classification and gene-sharing analysis) and updated the Figure 2 and the legend on lines 284-299 and below.

Figure 2 Legend lines 284-299: “Fig 2. Supervised machine learning prediction of antimicrobial resistance signature profiles to 21 antimicrobials in the E. coli cohort. A) Flow diagram showing the machine learning pipeline including feature selection (orange), classification (blue), post-processing (green); B) Prediction performance results of the RBF SVM classifier that achieved the best performance amongst the three investigated is shown. Five performance indicators have been used to evaluate the classification: i) Area under the curve AUC, ii) Accuracy, iii) Sensitivity, iv) Precision, v) Specificity, and vi) Cohen’s Kappa value from 30 training runs for each antimicrobial. The scores for each performance metric are indicated in the Y axis. Predictive models were generated to classify the resistance vs. susceptibility profiles of twenty-one different antimicrobials (X axis): amoxycillin/clavulanic acid (AMC), ampicillin (AMP), ampicillin/sulbactam (AMS), aztreonam (AZM), ceftazidime (CAZ), cefoxitin (CFX), cefazolin (CFZ), chloramphenicol (CHL), ciprofloxacin (CIP), cefotaxime (CTX), doxycycline (DOX), cefepime (FEP), gentamicin (GEN), kanamycin (KAN), levofloxacin (LEV), minocycline (MIN), nalidixic acid (NAL), streptomycin (STR), trimethoprim/sulfamethoxazole (SXT), sulfisoxazole (SUL) and tetracycline (TET).”

8. Why did the authors only test 3 machine learning models? Other advanced algorithms should be assessed also.

The number and types of machine learning algorithms were carefully considered when developing our analysis pipelines. A review of literature in this subject area showed many published works with three or fewer classifiers used, including our own previous works (Pearcy *et al.* 2021, Wang *et al.* 2021), also see Benkwitz-Bedford *et al.* 2021, Hicks *et al.* 2019, Nguyen *et al.* 2018, Maguire *et al.* 2019, ValizadehAslani *et al.* 2020, Aytan-Aktug *et al.* 2020, Liu *et al.* 2020, and Kavvas *et al.* 2018. In addition, when developing our pipelines for earlier works we used a larger panel of machine learning classifiers but found that they all gave fairly similar results, so in the interests of a concise and interesting paper we selected the three that were marginally better.

However, we acknowledge the reviewer’s comment and that also other readers may be interested in the performance of a wider range of classifiers and so we have now tested our models against 7 further classifiers, specifically: Extra Tree Classifier, Random Forest, Adaboost, Gradient Boosting Machine (XGBoost), Naïve Bayes, Linear Discriminant Analysis (LDA) and Gaussian Discriminant Analysis (GDA) and added the results of these further analyses to Supplementary Table 2 and Supplementary Figure 2. The results on lines 252-259 and 266-279 and methods on lines 1067-1071 and 1097-1105 were also updated to reflect the inclusion of these methods. We feel that the reviewer’s suggestion has improved the integrity and the results of our manuscript and we thank the reviewer for this very useful suggestion.

Also, to further reflect the reviewer’s comment we included a statistical analysis based on the Friedman Statistical F-test to compare the results for the 10 classifiers studied. The Friedman test F_F_ with Iman-Davenport correction (Iman & Davenport, 1980) is employed for statistical comparison of multiple classifiers as suggested by Demsar *et al.* (2006). First, we rank the algorithms for each dataset separately, i.e. the best algorithm gets ranking 1, the second best ranking 2, and so on. In case of ties, average ranks are assigned. After, the post-hoc Nemenyi test (Demsar *et al.* 2006) was used to find if there is a single classifier or a group of classifiers that differ in terms of their average rank after the F_F_ test has rejected the null hypothesis that the performance of the comparisons on the groups of data is similar.

With 10 classifiers and 21 antibiotics, the Friedman test is distributed according to the F distribution with 21−1 = 20 and (10−1)×(21−1) = 180 degrees of freedom. The critical value of F(9,180) for α= 0.0001 is 4.0366. For the metrics studies (AUC, accuracy, sensitivity, specificity, precision and Cohen’s Kappa), the F_F_ statistics null hypothesis was rejected with a confidence level of 99.99%. The null hypothesis states that there are no differences between the average ranks of each classifier over the 21 antibiotics. After, the Nemenyi post-hoc test was performed, and the critical difference diagram was set at 3.407 with a confidence level of 99%. The classifier RBF-SVM had the best rank for the metrics AUC, accuracy, sensitivity, Cohen’s kappa, second best for precision and third best for specificity. Nonetheless, according to the Nemenyi test, for precision and specificity, RBF-SVM is statistically equivalent when compared to the best ranked classifier in these two metrics. On the Nemenyi diagrams below it is possible to see that the three classifiers initially chosen are always statistically equivalent to the best ranked classifier. Hence, we have chosen to continue to use the RBF-SVM classifier results for our analysis.

Supplementary Figure 2. Nemenyi Diagram. Nemenyi post-hoc test for the performance metrics (A) AUC, (B) accuracy, (C) sensitivity, (D) specificity, (E) precision and (F) Cohen’s Kappa score for the following classifiers: logistic regression, linear SVM, RBF-SVM, extra tree classifier, random forest, adaboost, xgboost, naïve bayes, linear discriminant analysis (LDA) and quadratic discriminant analysis (QDA).

We have now included additional explanation of this analysis in the Methods on lines 1067-1071 and 1097-1105, updated the results section on lines 252-259 and lines 266-279, and included the results on Supplementary Table 2 and Supplementary Figure 2 and below:

Results lines 252-259: “Ten supervised learning classifiers, logistic regression, linear support vector machine, radial basis function kernel support vector machine (RBF-SVM), extra tree classifier, random forest, adaboost, xgboost, naïve bayes, linear discriminant analysis and quadratic discriminant analysis were used to predict susceptible and resistant strains for each of the 26 antimicrobials. All genome sequences were processed to give overlapping 13 base pair k-mer features related to each sequence. Five antibiotics (amikacin, polymyxin B, polymyxin E, imipenem and meropenem) did not have enough samples in one class to allow cross validation and SMOTE and so were not taken further.”

Results Lines 266-279: “To verify which classifier performs better out of the 10 classifiers studied we applied a Friedman F-test with Nemenyi post hoc test. With 10 classifiers and 21 antibiotics, the Friedman test is distributed according to the F distribution with 21−1 = 20 and (10−1)×(21−1) = 180 degrees of freedom. The critical value of F(9,180) for α= 0.0001 is 4.0366. For all the metrics studies (AUC, accuracy, sensitivity, specificity, precision and Cohen’s Kappa), the F_F_ statistics null hypothesis was rejected with a confidence level of 99.99%. The null hypothesis states that there are no differences between the average ranks of each classifier over the 21 antibiotics. After, the Nemenyi post-hoc test was performed, and the critical difference diagram was set at 3.407 with a confidence level of 99%. According to Supplementary Figure 2, the classifier RBF-SVM had the best rank for the metrics AUC, accuracy, sensitivity, Cohen’s kappa, second best for precision and third best for specificity. Nonetheless, according to the Nemenyi test, for precision and specificity, RBF-SVM is statistically equivalent when compared to the best ranked classifier in these two metrics. Therefore, the RBF-SVM classifier was selected for this study.”

Methods lines 1067-1071: “A panel of machine learning methods (logistic regression (LR), linear support vector machine (L-SVM), and radial basis function support vector machine (RBF-SVM), extra tree classifier, random forest, adaboost, xgboost, naïve bayes, linear discriminant analysis and quadratic discriminant analysis) were then run using as input the k-mers uncovered on the first step and their performances were evaluated.”

Methods Lines 1097-1105: “To compare the results obtained by the 10 different classifiers used, a Friedman Statistical F-test was employed. The Friedman test F_F_ with Iman-Davenport correction (Iman & Davenport, 1980) is employed for statistical comparison of multiple classifiers as suggested by Demsar *et al.* (2006). First, we rank the algorithms for each dataset separately, i.e., the best algorithm gets ranking 1, the second-best ranking 2, and so on. In case of ties, average ranks are assigned. Next, we apply the F_F_ and verify if the null hypothesis is rejected. After, the post-hoc Nemenyi test (Demsar *et al.* 2006) was used to find if there is a single classifier or a group of classifiers that differ in terms of their average rank after the F_F_ test has rejected the null hypothesis that the performance of the comparisons on the groups of data is similar.”

Supplementary Figure 2 lines 1602-1606: “Supplementary Figure 2. Nemenyi Diagram. Nemenyi post-hoc test for the performance metrics (A) AUC, (B) accuracy, (C) sensitivity, (D) specificity, (E) precision and (F) Cohen’s Kappa score for the following classifiers: logistic regression, linear SVM, RBF-SVM, extra tree classifier, random forest, adaboost, xgboost, naïve bayes, linear discriminant analysis (LDA) and quadratic discriminant analysis (QDA).”

Supplementary Table 2 lines 1670-1676: “Supplementary Table 2. Performance metrics (AUC, Accuracy, Sensitivity, Specificity, and Cohen’s Kappa value) results for the RBF-SVM classification into resistance or susceptible for 21 antibiotics and for the other 9 classifiers used (logistic regression, linear SVM, extra tree classifier, random forest, adaboost, xgboost, naïve bayes, linear discriminant analysis and quadratic discriminant analysis); Genes in which k-mers selected as features for the machine learning were found; Jaccard coefficients indicating the gene co-occurrence for all genes found to be significantly correlated with AMR phenotypes.”

Aytan-Aktug D, Clausen PTLC, Bortolaia V, Aarestrup FM, Lund O, Marshall CW. Prediction of Acquired Antimicrobial Resistance for Multiple Bacterial Species Using Neural Networks. mSystems. 2020;5(1):e00774-19.

Benkwitz-Bedford S, Palm M, Demirtas TY, Mustonen V, Farewell A, Warringer J, et al. Machine Learning Prediction of Resistance to Subinhibitory Antimicrobial Concentrations from Escherichia coli Genomes. mSystems. 2021;6(4):e00346-21.Hicks AL, Wheeler N, Sánchez-Busó L, Rakeman JL, Harris SR, Grad YH. Evaluation of parameters affecting performance and reliability of machine learning-based antibiotic susceptibility testing from whole genome sequencing data. PLOS Computational Biology. 2019;15(9):e1007349.

Demsar, J. Statistical comparisons of classifiers over multiple data sets, J. Mach. Learn. Res., vol. 7, pp. 1–30, Dec. 2006, ISSN: 1532-4435

Iman, R. L. and Davenport, J. M. Approximations of the critical region of the fbietkan statistic, Communications in Statistics - Theory and Methods, vol. 9,no. 6, pp. 571–595, 1980

Kavvas ES, Catoiu E, Mih N, Yurkovich JT, Seif Y, Dillon N, et al. Machine learning and structural analysis of Mycobacterium tuberculosis pan-genome identifies genetic signatures of antibiotic resistance. Nature Communications. 2018;9(1):4306.

Liu Z, Deng D, Lu H, Sun J, Lv L, Li S, et al. Evaluation of Machine Learning Models for Predicting Antimicrobial Resistance of Actinobacillus pleuropneumoniae From Whole Genome Sequences. Frontiers in Microbiology. 2020;11.

Maguire F, Rehman MA, Carrillo C, Diarra MS, Beiko RG. Identification of Primary Antimicrobial Resistance Drivers in Agricultural Nontyphoidal Salmonella enterica Serovars by Using Machine Learning. mSystems. 2019;4(4):e00211-19.

Nguyen M, Long SW, McDermott PF, Olsen RJ, Olson R, Stevens RL, et al. Using machine learning to predict antimicrobial MICs and associated genomic features for nontyphoidal Salmonella. Journal of clinical microbiology. 2019;57(2):e01260-18.

Pearcy N, Hu Y, Baker M, Maciel-Guerra A, Xue N, Wang W, et al. Genome-Scale Metabolic Models and Machine Learning Reveal Genetic Determinants of Antibiotic Resistance in Escherichia coli and Unravel the Underlying Metabolic Adaptation Mechanisms. mSystems. 2021;6(4):e00913-20.

ValizadehAslani T, Zhao Z, Sokhansanj BA, Rosen GL. Amino Acid k-mer Feature Extraction for Quantitative Antimicrobial Resistance (AMR) Prediction by Machine Learning and Model Interpretation for Biological Insights. Biology. 2020;9(11):365.

Wang W, Baker M, Hu Y, Xu J, Yang D, Maciel-Guerra A, et al. Whole-Genome Sequencing and Machine Learning Analysis of Staphylococcus aureus from Multiple Heterogeneous Sources in China Reveals Common Genetic Traits of Antimicrobial Resistance. mSystems. 2021;6(3):e01185-20.

**Reviewer #2**

The overall idea of the study is good, and the objectives are clear. However, I have some significant concerns.

We thank and are pleased that the reviewer appreciated the study idea is good and felt the objectives were clear.

1) The study size is very small. It was tough for me to aggregate all the relevant information. Please add a table (not as a supplement) where you put the number of samples for each drug, i.e., the counts of resistant and susceptible samples per drug.

We apologise if the data we presented was not clear and thank the reviewer for the suggestion. The number of resistant and susceptible samples per drug were presented graphically in Fig 1c, however, for clarity we have now also added this as a separate table (Table 1) as suggested by the reviewer. Table 1 is on line 197 in the main manuscript, see line 197 and below.

Table 1: Number of resistant and susceptible isolates included in this study.

| **Antibiotic** | **Abbreviation** | **Number of Resistant Isolates** | **Number of Susceptible Isolates** |
| --- | --- | --- | --- |
| Amikacin | AMI | 9 | 145 |
| Amoxycillin/Clavulanic acid | AMC | 26 | 54 |
| Ampicillin | AMP | 134 | 20 |
| Ampicillin/Sulbactam | AMS | 82 | 30 |
| Aztreonam | AZM | 74 | 68 |
| Cefazolin | CFZ | 108 | 27 |
| Cefepime | FEP | 62 | 66 |
| Cefotaxime | CTX | 92 | 60 |
| Cefoxitin | CFX | 15 | 133 |
| Ceftazidime | CAZ | 30 | 106 |
| Chloramphenicol | CHL | 107 | 41 |
| Ciprofloxacin | CIP | 93 | 48 |
| Colistin | CT | 8 | 146 |
| Doxycycline | DOX | 91 | 20 |
| Gentamicin | GEN | 51 | 76 |
| Imipenem | IMI | 5 | 144 |
| Kanamycin | KAN | 89 | 63 |
| Levofloxacin | LEV | 96 | 40 |
| Meropenem | MEM | 5 | 146 |
| Minoocycline | MIN | 16 | 109 |
| Nalidixic acid | NAL | 118 | 36 |
| Polymyxin B | PB | 6 | 147 |
| Streptomycin | STR | 117 | 37 |
| Sulphafurazole | SUL | 137 | 17 |
| Tetracycline | TET | 130 | 19 |
| Trimethoprim/Sulfamethoxazole | SXT | 132 | 22 |

We thank the reviewer for drawing our attention the important limitation of small sample sizes that is present in both ours and many other similar studies in this field. We acknowledge that the sample size is relatively small given the number of features. However, given the large number of features inherently present in omics data as well as the limitations on sample size (of limited *in vivo* experiments, protocols, involvement of human participants and costs) a large observations-features ratio is very often present in studies of this type. Many other published studies have used similar or lower numbers of isolates with good model performance, see Her and Wu 2018 (59 observations), Maguire 2019 (97 observations), Pincus 2020 (115 observations), Liu 2020 (96 observations). Further, a requirement of larger sample sizes would impose limitations on data availability, often requiring reliance on public databases and thus compromise on the type and integrity of available data and possible studies.

Finally, we would argue that the nested cross validation method we employed has been shown to be robust for small sample sizes (Vabalas *et al.* 2019) and in fact the machine learning performance metrics we have achieved for many antibiotics show that this is indeed the case. Our AUC values ranged from 0.906 ± 0.01 to 0.999 ± 0.002 with 9 models having a mean AUC of over 0.97. Similarly, the precision was also high with values ranging from 0.706 ± 0.107 to 0.991 ± 0.03 and 11 models having a mean precision over 0.94. Comparing this performance with studies with larger datasets as for example in Ren 2021 with two independent datasets tested against 4 antibiotics with no less than 900 observations per model, the AUC in our smaller dataset was higher in 7 of the 8 models, and comparable with the 8^th^ model (gentamicin prediction using Giessen dataset). In terms of recall/sensitivity, our smaller dataset outperformed all 6 of the 8 models. Similarly, in Moradigaravand, 2018 of 6 comparable antibiotics our smaller dataset had the same or better sensitivity (R.RCL) in 5 of 6 models and the same or better specificity (S.RCL) in 3 of 6 models.

However, to reflect the reviewer’s comment we have performed further analyses, to test if the dataset of each antibiotic and hence the sample size used for each antibiotic model is representative. Specifically, we have created multiple learning curves to compare the performance on the training and test sets of the outer loop in the nested cross validation. An unrepresentative dataset means a dataset that does not capture the statistical characteristics relative to another dataset drawn from the same domain, such as training and test folds. This will commonly occur if the dataset is too small (Brownlee, 2019).

Learning curves show the model performance as a function of the number of samples and can help determine the sample size needed to train a good classifier (Beleites *et al.*, 2013). When comparing the performance function of the training fold with the test fold it is possible to evaluate if we are undertraining, overtraining or have a good performance. Undertraining refers to a model that cannot learn the training set. Overfitting refers to a model that has learned the training dataset too well, but when applied to the test fold the performance decreases dramatically creating a large gap between the training and testing performances; this indicates that the test fold is unrepresentative of the training fold. A good fit learning curve is the goal of all the algorithms and is represented by a small gap between the training and the testing curves (Beleites *et al.*, 2013).

To analyse the 21 different antibiotics in this study we used a wrapper backward selection (WBS) approach in terms of samples, i.e. if initially we have 100 samples we are first going to test the model using all 99 possible sample combinations (leaving one out), the worst performance is identified and the sample that was left out on this performance is removed for the next iteration. We do this until the minimum number of samples (12) is reached for one of the classes. This minimum number of samples is a requirement for the SMOTE approach used in our framework. The further test if the sample sizes used in this work were representative we applied the WBS approach to two analyses and results compared: firstly, we applied the WBS (see Supplementary Figure 3A), to the original pipeline used in this work (i.e. nested cross validation with an RBF-SVM as the classifier and a SMOTE approach during the training phase to balance the classes for the training of the classifier (as used in our ML pipeline), 5 iterations of the nested cross validation were used (see updated methods regarding the explanation of the nested cross validation)). While, for the second analysis, we first performed a SMOTE approach as a pre-processing step to oversample the minority class and then applied the WBS method on the oversampled data, and analysed using a nested cross validation with a RBF-SVM as the classifier (Supplementary Figure 3B). The SMOTE step during the training phase was not used here, since the class were balanced as a pre-processing step. For the second analysis we have also used 5 iterations of the nested cross-validation. The features used in the two analyses were the same and are the ones described in the manuscript for each antibiotic, with their performance indicated on Figure 2 and Supplementary Table 2 of the manuscript.

The results obtained for the first analysis are shown in Supplementary Figure 3A for 21 antibiotics. In 4 of 21 antibiotics (AMP, TET, CFX and MIN) the WBS finished the learning after a few iterations due to the small number of samples in the minority class. Nonetheless, according to the learning curves shown for 17 of 21 antibiotics in Supplementary Figure 3A, the samples used in the training and test datasets, for each antibiotic model, are representative (right side of the plot); Notably, the curve of the test performance for all models is reaching an asymptote indicating that increasing sample sizes further would be unlikely to significantly improve model performance.

However, if the number of samples are reduced from the original one used to develop the models, the majority of the antibiotics classifiers decrease in the test fold performance, indicating that a smaller number of samples with respect to what used in this work would cause an overfitting of the classifier, since the test fold is not representative anymore.

Notably, the results shown in Supplementary Figure 3B, obtained by creating synthetic data for the minority class during the pre-processing, reveal similar results as the ones obtained on Supplementary Figure 3A. In particular, the vertical blue line in each plot shows the original number of samples for each antibiotic. The shape of the learning curves is kept similar between Supplementary Figures 3A and 3B and it confirms that for all the antibiotics an increase in the number of samples would not have a big impact in the performance when compared to the original number of samples, which indicates that the features selected for each antibiotic are representative. This result also indicates that the performance of the RBF-SVM is robust with the number of samples we have used in each antibiotic model; this is likely due to the fact that we have used the nested cross validation. According to Vabalas *et al.,* 2019 the nested cross validation approach can give an unbiased performance estimate regardless of the sample size. Vabalas and collaborators indicated that by using a nested cross validation approach the models are able to learn patterns in the data more efficiently and hence can achieve high performance results. In their work, they concluded that separating completely testing and training data is enough to obtain unbiased performance estimates regardless of sample size.

Supplementary Figure 3. Learning Curves. Learning curves of the 21 antibiotics using a wrapper backward selection to evaluate the training and testing performance whilst decreasing the number of samples. (A) The original number of samples for each antibiotic and 5 nested cross validation iterations with an RBF-SVM as the main classifier; (B) SMOTE approach as a pre-processing step to increase the number of samples by adding synthetic samples to the minority class for each antibiotic and 5 nested cross validation iterations with an RBF-SVM as the main classifier. In both cases, the same features used to acquire the performances in the Figure 2 and Supplementary Table 2 are used and kept for all the iterations of the WBS. The red lines indicate the training performance, the green lines the testing performance and the blue vertical line the original number of samples.

We thank the reviewer for his/her suggestion regarding justifying the sample size used in this work and we feel that the analysis we did to address his/her comment improved the quality of the manuscript. We have now updated the manuscript see results lines 309-332, discussion lines 681-762 and methods lines 1107-1113 and Supplementary Figure 3 in lines 1608-1617 and below:

Results Lines 309-332: “To verify if the number of samples for each antibiotic model was large enough for the test set to be representative, we employed a wrapper backward selection (WBS) in terms of the samples using two different analyses: (i) firstly, we applied the WBS (see Supplementary Figure 3A), to the original pipeline used in this work (i.e. nested cross validation with an RBF-SVM as the classifier and a SMOTE approach during the training phase to balance the classes for the training of the classifier (as used in our ML pipeline), 5 iterations of the nested cross validation were used; (ii) while, for the second analysis, we first performed a SMOTE approach as a pre-processing step to oversample the minority class and then applied the WBS method on the oversampled data, and analysed using 5 iterations of a nested cross validation with a RBF-SVM as the classifier (Supplementary Figure 3B). Supplementary Figure 3A shows that for 4 out of 21 antibiotics (AMP, TET, CFX and MIN) the WBS finished after a few iterations due to the small number of samples in the minority class. Nonetheless, according to the learning curves of the other 17 out of 21 antibiotics on Supplementary Figure 3A the training and test datasets are representative for the initial number of samples in each antibiotic; with a reduced number of samples the majority of the antibiotics have a decrease in the test fold performance, indicating that a reduced number of samples in this case can cause an overfitting of the classifier, since the test fold is not representative anymore. The curve of the test performance for all models is reaching an asymptote indicating that increasing sample sizes further would be unlikely to significantly improve model performance. Moreover, on Supplementary Figure 3B, which creates synthetic data for the minority class during the pre-processing, reveals a similar result to the first approach in Supplementary Figure 3A. The shape of the learning curves is kept similar between the two studies and it confirms for all the antibiotics that an increase in the number of samples would not have a big impact in the performance when compared to the original number of samples.”

Discussion lines 681-762: “A recent study by Ren (Ren *et al.* 2021) also looked at prediction of *E. coli* resistance from human and animal isolates from two datasets, one they term the Giessen data, 987 whole genome *E. coli* sequences they collected as part of their study and a public dataset of 1509 *E. coli* strains taken from Moradigaravand et al. (2018). Ren tested both of these datasets against four antibiotics (ciprofloxacin, cefotaxime, ceftazidime and gentamicin) all of which we also tested allowing comparison between our study and theirs. There were several key differences between our study and theirs: the Giessen data in Ren are described as human and animal clinical samples with no source country given and the public data were retrieved from both human clinical and environmental collections in the UK, Pakistan, Syria, Sweden, the USA, and Belgium. In contrast our data were from interconnected human, animal and environmental sources on a single Chinese poultry farm and connected slaughterhouse. Ren used SNPs (called against *E. coli* K-12 reference) as input to their classifiers, with no feature selection, whereas we used k-mers and performed a two-step feature selection to reduce sample-feature ratio. Ren then used Logistic regression, linear SVM, Random Forest and convolutional neural network classifiers to predict AMR phenotypes. In our study we used 10 classifiers including 3 that Ren used (LR, SVM, RF). Comparing the four antibiotics models across the two studies, our machine learning models achieved equal or higher AUC and precision values compared to all four antibiotic models. The sensitivity of our models was the same or better compared to both the Giessen and public datasets for all antibiotics except ceftazidime, where our sensitivity was 0.81 compared to 0.74-0.90 in the Ren study. For each of their datasets, Ren took the 10 highest-ranking features from each antibiotic and associated them with the genes in the genome to identify the cognate markers of AMR. Excluding known AMR genes, they found 19 genes of interest. Interestingly, 14 out of these 19 genes (*nha*A, *rlm*C, *fli*I, *pep*B, *prl*C, *sod*A, *mur*B, *rlu*F, *yjf*F, *tre*R, *arg*I, *val*S, *fhu*F and *nad*R) were also selected by our machine learning algorithms, and 4 (*sod*A, *rlu*F, *tre*R and *arg*I) were found to be in the 361 most discriminating genes. This overlapping of genes despite the different sample sources and countries (predominantly clinical isolates from humans and animals of international origin, compared to isolates from healthy animals and humans from China in our study) gives further evidence of the robustness of the methods we have used in this study.

Despite the limited number of samples available in our study, when we compared our machine learning performance metrics to published studies with similar and larger *E. coli* datasets we found comparable AUC, sensitivity, and specificity values (Her and Wu 2018, Moradigaravand *et al.* 2018, Hyun *et al.* 2020, Ren *et al.* 2021), indicating that the methods employed in this study have not been excessively affected by this limitation. Specifically, Hyun (Hyun *et al.* 2020) used 1588 *E. coli* genomes from publicly available data to build SVM-based prediction models using amino acid sequence variants. The genomes used were of human origin and predominantly from the UK and USA. Hyun tested six antibiotics of which four overlapped with those in our study (AMC, CAZ, CIP and GEN). Compared to Hyun across these 4 antibiotics we achieved better AUC values in our study for 3 of 4 models (AMC: 0.83 vs 0.91, CAZ: 0.95 vs 0.97, GEN: 0.94 vs 0.95 [Hyun vs ours]), with our AUC for CIP slightly worse with a value of 0.97 compared to 0.98. Our sensitivity (recall) was better than in Hyun for all 4 antibiotics. Our models performed slightly worse against Hyun in three antibiotics for accuracy (CAZ: 0.97 vs 0.92, CIP: 0.98 vs 0.94, GEN: 0.97 vs 0.90 [Hyun vs ours]) and precision (CAZ: 0.87 vs 0.88, CIP: 0.94 vs 0.96, GEN: 0.86 vs 0.94 [Hyun vs ours]). However, our AMC model performed better for both precision (0.67 vs 0.80) and accuracy (0.79 vs 0.85).

Similarly, in Moradigaravand (Moradigaravand *et* *al.* 2018) 1936 *E. coli* sequences (of which 1509 were also used in Ren, 2018) from human clinical and environmental sources were retrieved from collections in the UK, Pakistan, Syria, Sweden, the USA, and Belgium and used to predict AMR phenotypes using accessory gene presence-absence, matrix of the population structure, core genome SNPs, matrix of indels (insertions and deletions) and the year of isolation as features. Four machine learning classifiers were considered Random Forest, Gradient boosted decision trees, deep neural networks and logistic regression. Eleven antibiotics models were considered in Moradigaravand of which 6 overlapped with those of our study: AMC, AMP, CIP, CTX, CAZ (denoted CTZ in Moradigaravand) and GEN. Of these 6 antibiotics our dataset had better accuracy in 3 of 6 models (AMC: 0.81 vs 0.88, AMP: 0.93 vs 0.98, CIP 0.93 vs 0.94, CTX 0.97 vs 0.89, CAZ: 0.95 vs 0.93, and GEN: 0.97 vs 0.90 [Moradigaravand vs ours]). Our study had the same or better sensitivity (denoted R.RCL in (Moradigaravand *et al.* 2018)) in 5 of the 6 models (AMC: 0.64 vs 0.89, AMP: 0.96 vs 0.98, CIP 0.81 vs 0.97, CTX 0.92 vs 0.91, CAZ: 0.80 vs 0.80, and GEN: 0.81 vs 0.91 [Moradigaravand vs ours]). Comparing specificity our study achieved same or better specificity (denoted S.RCL in (Moradigaravand *et al*. 2018)) in 3 of 6 models (AMC: 0.60 vs 0.88, AMP: 0.96 vs 0.94, CIP 0.87 vs 0.88, CTX 0.93 vs 0.87, CAZ: 0.92 vs 0.96, and GEN: 0.98 vs 0.89 [Moradigaravand vs ours]).

Another *E coli* study with a smaller sample size compared to ours was that of Her and Wu (Her and Wu 2018). Her took 59 public database sequences (51 USA human isolates, 1 Taiwan human isolate, 1 Brazilian penguin isolate and 6 of unknown origin) and built machine learning predictors using radial SVM, Naïve Bayes, Adaboost, and Random Forest classifiers. Four different feature sets were used: core + accessory gene clusters, accessory gene clusters, CARD gene clusters and accessory gene cluster with CARD annotation. Of these the core + accessory gene cluster feature set is the most comparable with our k-mer features that cover both the core and accessory genome. Her and Wu studied 12 antibiotics of which eight overlapped with those studied in our work (AMP, AMS, CFZ, CAZ, FEP, GEN, CIP and SXT). Compared to Her and Wu (Her and Wu 2018) (looking at the most comparable feature set core + accessory gene clusters) our model gave the same or better AUC values for all the classifiers used in Her (AMP: 0.75 vs 1.00, AMS: 0.70 vs 0.97, CAZ: 0.84 vs 0.97, CFZ: 0.82 vs 1.00, FEP: 0.85 vs 0.94, GEN: 0.72 vs 0.95, CIP: 0.78 vs 0.97 and SXT 0.66 vs 0.99 [Her vs Ours]). Additionally, we achieved equal or better sensitivity values across 5 of 8 antibiotics compared Her and Wu (AMP: 0.5 vs 0.98, AMS: 0.53 vs 0.99, CAZ: 0.80 vs 0.80, CFZ: 0.86 vs 0.98, FEP: 0.90 vs 0.89, GEN: 1.00 vs 0.91, CIP: 1.00 vs 0.97 and SXT 0.49 vs 0.97 [Her vs Ours]), and better precision also in 5 antibiotics (AMP: 1.00 vs 0.99, AMS: 1.00 vs 0.95, CAZ: 0.81 vs 0.87, CFZ: 0.79 vs 0.99, FEP: 0.82 vs 0.89, GEN: 0.85 vs 0.86, CIP: 0.73 vs 0.94 and SXT 1.00 vs 0.98 [Her vs Ours]).”

Method Lines 1107-1113: “To analyse if the antibiotics studied had enough samples to make the test representative, a wrapper backward selection (WBS) approach in terms of samples was used, i.e. if initially we have 100 samples we are first going to test the model using all 99 possible sample combinations (leaving one out), the worst performance is identified and the sample that was left out on this performance is removed for the next iteration. This is done until the minimum number of samples (12) is reached for one of the classes. This minimum number of samples is a requirement for the SMOTE approach used in the classification framework.”

Supplementary Figure 3 lines 1608-1617: “Supplementary Figure 3. Learning Curves. Learning curves of the 21 antibiotics using a wrapper backward selection to evaluate the training and testing performance whilst decreasing the number of samples. (A) The original number of samples for each antibiotic and 5 nested cross validation iterations with an RBF-SVM as the main classifier; (B) SMOTE approach as a pre-processing step to increase the number of samples by adding synthetic samples to the minority class for each antibiotic and 5 nested cross validation iterations with an RBF-SVM as the main classifier. In both cases, the same features used to acquire the performances in the Figure 2 and Supplementary Table 2 are used and kept for all the iterations of the WBS. The red lines indicate the training performance, the green lines the testing performance and the blue vertical line the original number of samples.”

Brownlee, J. (2019) “How to use Learning Curves to Diagnose Machine Learning Model Performance” Machine Learning Mastery. Accessed on January 28^th^ 2022. https://machinelearningmastery.com/learning-curves-for-diagnosing-machine-learning-model-performance/

Her, H. L. and Wu Y. W. (2018). "A pan-genome-based machine learning approach for predicting antimicrobial resistance activities of the Escherichia coli strains." Bioinformatics 34(13): i89-i95.

Hyun JC, Kavvas ES, Monk JM, Palsson BO. Machine learning with random subspace ensembles identifies antimicrobial resistance determinants from pan-genomes of three pathogens. PLOS Computational Biology. 2020;16(3):e1007608.

Liu, Z., et al. (2020). "Evaluation of Machine Learning Models for Predicting Antimicrobial Resistance of Actinobacillus pleuropneumoniae From Whole Genome Sequences." Frontiers in Microbiology 11.

Maguire, F., et al. (2019). "Identification of Primary Antimicrobial Resistance Drivers in Agricultural Nontyphoidal Salmonella enterica Serovars by Using Machine Learning." mSystems 4(4): e00211-00219.

Moradigaravand, D., et al. (2018). "Prediction of antibiotic resistance in Escherichia coli from large-scale pan-genome data." PloS Computational Biology 14(12): e1006258.

Pincus, N. B., et al. (2020). "A Genome-Based Model to Predict the Virulence of Pseudomonas aeruginosa Isolates." MBio 11(4): e01527-01520.

Ren, Y., et al. (2021). "Prediction of antimicrobial resistance based on whole-genome sequencing and machine learning." Bioinformatics (Oxford, England) 38(2): 325-334.

Vabalas A *et al.*, Machine learning algorithm validation with a limited sample size. PLoS One. 2019; 14(11): e0224365.

2) The machine learning approach is very similar to the recently published paper by Ren et al. (pubmed:34613360). However, they used fewer antibiotics but far more samples. Please discuss your findings in the context of this study and maybe check whether you found similar genetic information / SNPs.

We thank the reviewer for highlighting this recent publication that we had not included in our manuscript. Comparison with this study was valuable and provided further evidence of the good performance and robustness of our methods. We have added a comparison of the two papers on the discussion on lines 681-709 and below:

Discussion Lines 681-709: “A recent study by Ren (Ren *et al.* 2021) also looked at prediction of *E. coli* resistance from human and animal isolates from two datasets, one they term the Giessen data, 987 whole genome *E. coli* sequences they collected as part of their study and a public dataset of 1509 *E. coli* strains taken from Moradigaravand et al. (2018). Ren tested both of these datasets against four antibiotics (ciprofloxacin, cefotaxime, ceftazidime and gentamicin) all of which we also tested allowing comparison between our study and theirs. There were several key differences between our study and theirs: the Giessen data in Ren are described as human and animal clinical samples with no source country given and the public data were retrieved from both human clinical and environmental collections in the UK, Pakistan, Syria, Sweden, the USA, and Belgium. In contrast our data were from interconnected human, animal and environmental sources on a single Chinese poultry farm and connected slaughterhouse. Ren used SNPs (called against *E. coli* K-12 reference) as input to their classifiers, with no feature selection, whereas we used k-mers and performed a two-step feature selection to reduce sample-feature ratio. Ren then used Logistic regression, linear SVM, Random Forest and convolutional neural network classifiers to predict AMR phenotypes. In our study we used 10 classifiers including 3 that Ren used (LR, SVM, RF). Comparing the four antibiotics models across the two studies, our machine learning models achieved equal or higher AUC and precision values compared to all four antibiotic models. The sensitivity of our models was the same or better compared to both the Giessen and public datasets for all antibiotics except ceftazidime, where our sensitivity was 0.81 compared to 0.74-0.90 in the Ren study. For each of their datasets, Ren took the 10 highest-ranking features from each antibiotic and associated them with the genes in the genome to identify the cognate markers of AMR. Excluding known AMR genes, they found 19 genes of interest. Interestingly, 14 out of these 19 genes (*nha*A, *rlm*C, *fli*I, *pep*B, *prl*C, *sod*A, *mur*B, *rlu*F, *yjf*F, *tre*R, *arg*I, *val*S, *fhu*F and *nad*R) were also selected by our machine learning algorithms, and 4 (*sod*A, *rlu*F, *tre*R and *arg*I) were found to be in the 361 most discriminating genes. This overlapping of genes despite the different sample sources and countries (predominantly clinical isolates from humans and animals of international origin, compared to isolates from healthy animals and humans from China in our study) gives further evidence of the robustness of the methods we have used in this study.”

Ren, Y., et al. (2021). "Prediction of antimicrobial resistance based on whole-genome sequencing and machine learning." Bioinformatics (Oxford, England) 38(2): 325-334.

3) I doubt the statistical robustness of non-linear kernels here. The sample size is tiny (still unclear, see 1)), with a lot of features (here: SNPs) and many different antibiotics. These things make it very likely that the results are not reproducible.

We agree with the reviewer’s comment, that the data sets used in this work have indeed a low number of samples, nonetheless, as discussed in the previous question, many other published studies have used similar or lower numbers of isolates with good model performance, see Her and Wu 2018 (59 observations), Maguire 2019 (97 observations), Pincus 2020 (115 observations) and Liu 2020 (96 observations).

To confirm the robustness of our method we made comparison to other similar studies using machine learning classification to predict AMR in E coli. This showed our results were comparable to studies with both larger and smaller datasets and has been added to the discussion lines 681-762 and below:

Discussion lines 681-762: “A recent study by Ren (Ren *et al.* 2021) also looked at prediction of *E. coli* resistance from human and animal isolates from two datasets, one they term the Giessen data, 987 whole genome *E. coli* sequences they collected as part of their study and a public dataset of 1509 *E. coli* strains taken from Moradigaravand et al. (2018). Ren tested both of these datasets against four antibiotics (ciprofloxacin, cefotaxime, ceftazidime and gentamicin) all of which we also tested allowing comparison between our study and theirs. There were several key differences between our study and theirs: the Giessen data in Ren are described as human and animal clinical samples with no source country given and the public data were retrieved from both human clinical and environmental collections in the UK, Pakistan, Syria, Sweden, the USA, and Belgium. In contrast our data were from interconnected human, animal and environmental sources on a single Chinese poultry farm and connected slaughterhouse. Ren used SNPs (called against *E. coli* K-12 reference) as input to their classifiers, with no feature selection, whereas we used k-mers and performed a two-step feature selection to reduce sample-feature ratio. Ren then used Logistic regression, linear SVM, Random Forest and convolutional neural network classifiers to predict AMR phenotypes. In our study we used 10 classifiers including 3 that Ren used (LR, SVM, RF). Comparing the four antibiotics models across the two studies, our machine learning models achieved equal or higher AUC and precision values compared to all four antibiotic models. The sensitivity of our models was the same or better compared to both the Giessen and public datasets for all antibiotics except ceftazidime, where our sensitivity was 0.81 compared to 0.74-0.90 in the Ren study. For each of their datasets, Ren took the 10 highest-ranking features from each antibiotic and associated them with the genes in the genome to identify the cognate markers of AMR. Excluding known AMR genes, they found 19 genes of interest. Interestingly, 14 out of these 19 genes (*nha*A, *rlm*C, *fli*I, *pep*B, *prl*C, *sod*A, *mur*B, *rlu*F, *yjf*F, *tre*R, *arg*I, *val*S, *fhu*F and *nad*R) were also selected by our machine learning algorithms, and 4 (*sod*A, *rlu*F, *tre*R and *arg*I) were found to be in the 361 most discriminating genes. This overlapping of genes despite the different sample sources and countries (predominantly clinical isolates from humans and animals of international origin, compared to isolates from healthy animals and humans from China in our study) gives further evidence of the robustness of the methods we have used in this study.

Despite the limited number of samples available in our study, when we compared our machine learning performance metrics to published studies with similar and larger *E. coli* datasets we found comparable AUC, sensitivity, and specificity values (Her and Wu 2018, Moradigaravand *et al.* 2018, Hyun *et al.* 2020, Ren *et al.* 2021), indicating that the methods employed in this study have not been excessively affected by this limitation. Specifically, Hyun (Hyun *et al.* 2020) used 1588 *E. coli* genomes from publicly available data to build SVM-based prediction models using amino acid sequence variants. The genomes used were of human origin and predominantly from the UK and USA. Hyun tested six antibiotics of which four overlapped with those in our study (AMC, CAZ, CIP and GEN). Compared to Hyun across these 4 antibiotics we achieved better AUC values in our study for 3 of 4 models (AMC: 0.83 vs 0.91, CAZ: 0.95 vs 0.97, GEN: 0.94 vs 0.95 [Hyun vs ours]), with our AUC for CIP slightly worse with a value of 0.97 compared to 0.98. Our sensitivity (recall) was better than in Hyun for all 4 antibiotics. Our models performed slightly worse against Hyun in three antibiotics for accuracy (CAZ: 0.97 vs 0.92, CIP: 0.98 vs 0.94, GEN: 0.97 vs 0.90 [Hyun vs ours]) and precision (CAZ: 0.87 vs 0.88, CIP: 0.94 vs 0.96, GEN: 0.86 vs 0.94 [Hyun vs ours]). However, our AMC model performed better for both precision (0.67 vs 0.80) and accuracy (0.79 vs 0.85).

Similarly, in Moradigaravand (Moradigaravand *et* *al.* 2018) 1936 *E. coli* sequences (of which 1509 were also used in Ren, 2018) from human clinical and environmental sources were retrieved from collections in the UK, Pakistan, Syria, Sweden, the USA, and Belgium and used to predict AMR phenotypes using accessory gene presence-absence, matrix of the population structure, core genome SNPs, matrix of indels (insertions and deletions) and the year of isolation as features. Four machine learning classifiers were considered Random Forest, Gradient boosted decision trees, deep neural networks and logistic regression. Eleven antibiotics models were considered in Moradigaravand of which 6 overlapped with those of our study: AMC, AMP, CIP, CTX, CAZ (denoted CTZ in Moradigaravand) and GEN. Of these 6 antibiotics our dataset had better accuracy in 3 of 6 models (AMC: 0.81 vs 0.88, AMP: 0.93 vs 0.98, CIP 0.93 vs 0.94, CTX 0.97 vs 0.89, CAZ: 0.95 vs 0.93, and GEN: 0.97 vs 0.90 [Moradigaravand vs ours]). Our study had the same or better sensitivity (denoted R.RCL in (Moradigaravand *et al.* 2018)) in 5 of the 6 models (AMC: 0.64 vs 0.89, AMP: 0.96 vs 0.98, CIP 0.81 vs 0.97, CTX 0.92 vs 0.91, CAZ: 0.80 vs 0.80, and GEN: 0.81 vs 0.91 [Moradigaravand vs ours]). Comparing specificity our study achieved same or better specificity (denoted S.RCL in (Moradigaravand *et al*. 2018)) in 3 of 6 models (AMC: 0.60 vs 0.88, AMP: 0.96 vs 0.94, CIP 0.87 vs 0.88, CTX 0.93 vs 0.87, CAZ: 0.92 vs 0.96, and GEN: 0.98 vs 0.89 [Moradigaravand vs ours]).

Another *E coli* study with a smaller sample size compared to ours was that of Her and Wu (Her and Wu 2018). Her took 59 public database sequences (51 USA human isolates, 1 Taiwan human isolate, 1 Brazilian penguin isolate and 6 of unknown origin) and built machine learning predictors using radial SVM, Naïve Bayes, Adaboost, and Random Forest classifiers. Four different feature sets were used: core + accessory gene clusters, accessory gene clusters, CARD gene clusters and accessory gene cluster with CARD annotation. Of these the core + accessory gene cluster feature set is the most comparable with our k-mer features that cover both the core and accessory genome. Her and Wu studied 12 antibiotics of which eight overlapped with those studied in our work (AMP, AMS, CFZ, CAZ, FEP, GEN, CIP and SXT). Compared to Her and Wu (Her and Wu 2018) (looking at the most comparable feature set core + accessory gene clusters) our model gave the same or better AUC values for all the classifiers used in Her (AMP: 0.75 vs 1.00, AMS: 0.70 vs 0.97, CAZ: 0.84 vs 0.97, CFZ: 0.82 vs 1.00, FEP: 0.85 vs 0.94, GEN: 0.72 vs 0.95, CIP: 0.78 vs 0.97 and SXT 0.66 vs 0.99 [Her vs Ours]). Additionally, we achieved equal or better sensitivity values across 5 of 8 antibiotics compared Her and Wu (AMP: 0.5 vs 0.98, AMS: 0.53 vs 0.99, CAZ: 0.80 vs 0.80, CFZ: 0.86 vs 0.98, FEP: 0.90 vs 0.89, GEN: 1.00 vs 0.91, CIP: 1.00 vs 0.97 and SXT 0.49 vs 0.97 [Her vs Ours]), and better precision also in 5 antibiotics (AMP: 1.00 vs 0.99, AMS: 1.00 vs 0.95, CAZ: 0.81 vs 0.87, CFZ: 0.79 vs 0.99, FEP: 0.82 vs 0.89, GEN: 0.85 vs 0.86, CIP: 0.73 vs 0.94 and SXT 1.00 vs 0.98 [Her vs Ours]).”

Moreover, to determine the robustness of non-linear kernels in our study, to reflect the reviewer’s comment we have included in the study an additional 7 classifiers (Extra Tree Classifier, Random Forest, Adaboost, Gradient Boosting Machine (XGBoost), Naïve Bayes, Linear Discriminant Analysis (LDA) and Gaussian Discriminant Analysis (GDA)) and compared them with the three classifiers already used (RBF-SVM, Linear SVM and Logistic Regression) using the Friedman F-test with a Nemeyi post-hoc test.

To further reflect the reviewer’s comment, we included a statistical analysis based on the Friedman Statistical F-test to compare the results for the 10 classifiers studied. The Friedman test F_F_ with Iman-Davenport correction (Iman & Davenport, 1980) is employed for statistical comparison of multiple classifiers as suggested by Demsar *et al.* (2006). First, we rank the algorithms for each dataset separately, i.e., the best algorithm gets ranking 1, the second best ranking 2, and so on. In case of ties, average ranks are assigned. After, the post-hoc Nemenyi test (Demsar *et al.* 2006) was used to find if there is a single classifier or a group of classifiers that differ in terms of their average rank after the F_F_ test has rejected the null hypothesis that the performance of the comparisons on the groups of data is similar.

With 10 classifiers and 21 antibiotics, the Friedman test is distributed according to the F distribution with 21−1 = 20 and (10−1)×(21−1) = 180 degrees of freedom. The critical value of F(9,180) for α= 0.0001 is 4.0366. For the metrics studies (AUC, accuracy, sensitivity, specificity, precision and Cohen’s Kappa), the F_F_ statistics null hypothesis was rejected with a confidence level of 99.99%. The null hypothesis states that there are no differences between the average ranks of each classifier over the 21 antibiotics. After, the Nemenyi post-hoc test was performed, and the critical difference diagram was set at 3.407 with a confidence level of 99%. The classifier RBF-SVM had the best rank for the metrics AUC, accuracy, sensitivity, Cohen’s kappa, second best for precision and third best for specificity. Nonetheless, according to the Nemenyi test, for precision and specificity, RBF-SVM is statistically equivalent when compared to the best ranked classifier in these two metrics.

Supplementary Figure 2. Nemenyi Diagram. Nemenyi post-hoc test for the performance metrics (A) AUC, (B) accuracy, (C) sensitivity, (D) specificity, (E) precision and (F) Cohen’s Kappa score for the following classifiers: logistic regression, linear SVM, RBF-SVM, extra tree classifier, random forest, adaboost, xgboost, naïve bayes, linear discriminant analysis (LDA) and quadratic discriminant analysis (QDA).

Moreover, in light of the reviewers’ concerns regarding the size of the dataset, to diagnose if the dataset of each antibiotic is representative, we have created multiple learning curves to compare the performance on the training and test sets of the outer loop in the nested cross validation. An unrepresentative dataset means a dataset that does not capture the statistical characteristics relative to another dataset drawn from the same domain, such as training and test folds. This will commonly occur if the dataset is too small (Brownlee, 2019).

Learning curves show the model performance as a function of the number of samples and can help determine the sample size needed to train a good classifier (Beleites *et al.*, 2013). When comparing the performance function of the training fold with the test fold it is possible to evaluate if we are undertraining, overtraining or have a good performance. Undertraining refers to a model that cannot learn the training set. Overfitting refers to a model that has learned the training dataset too well, but when applied to the test fold the performance decreases dramatically creating a large gap between the training and testing performances; this indicates that the test fold is unrepresentative of the training fold. A good fit learning curve is the goal of all the algorithms and is represented by a small gap between the training and the testing curves (Beleites *et al.*, 2013).

To analyse the 21 different antibiotics in this study we used a wrapper backward selection (WBS) approach in terms of samples, i.e. if initially we have 100 samples we are first going to test the model using all 99 possible sample combinations (leaving one out), the worst performance is identified and the sample that was left out on this performance is removed for the next iteration. We do this until the minimum number of samples (12) is reached for one of the classes. This minimum number of samples is a requirement for the SMOTE approach used in our framework. The further test if the sample sizes used in this work were representative we applied the WBS approach two analyses and results compared: (i) firstly, we applied the WBS (see Supplementary Figure 3A), to the pipeline used in this work: nested cross validation with an RBF-SVM as the classifier and a SMOTE approach during the training phase to balance the classes for the training of the classifier (as used in our ML pipeline), 5 iterations of the nested cross validation were used (see updated methods regarding the explanation of the nested cross validation); (ii) secondly, we applied a SMOTE approach as a pre-processing step to oversample the minority class and then applied the WBS method on the oversampled data, and analysed using a nested cross validation with a RBF-SVM as the classifier (Supplementary Figure 3B). The SMOTE step during the training phase was not used here, since the class were balanced as a pre-processing step. For the second analysis we have also used 5 iterations of the nested cross-validation. The features used with both approaches were the same ones found in the manuscript for each antibiotic, with their performance indicated on Figure 2 and Supplementary Table 2 of the manuscript.

The results obtained for the first analysis are shown in Supplementary Figure 3A for 21 antibiotics. In 4 of 21 antibiotics (AMP, TET, CFX and MIN) the WBS finished the learning after a few iterations due to the small number of samples in the minority class. Nonetheless, according to the learning curves shown for 17 of 21 antibiotics in Supplementary Figure 3A, the samples used in the training and test datasets, for each antibiotic model, are representative (right side of the plot); Notably, the curve of the test performance for all models is reaching an asymptote indicating that increasing sample sizes further would be unlikely to significantly improve model performance.

However, if the number of samples are reduced from the original one used to develop the models, the majority of the antibiotics classifiers decrease in the test fold performance, indicating that a smaller number of samples with respect to what used in this work would cause an overfitting of the classifier, since the test fold is not representative anymore.

Notably, the results shown in Supplementary Figure 3B, obtained by creating synthetic data for the minority class during the pre-processing, reveal similar results as the ones obtained on Supplementary Figure 3A. In particular, the vertical blue line in each plot shows the original number of samples for each antibiotic. The shape of the learning curves is kept similar between Supplementary Figures 3A and 3B and it confirms that for all the antibiotics an increase in the number of samples would not have a big impact in the performance when compared to the original number of samples, which indicates that the features selected for each antibiotic are representative. This result also indicates that the performance of the RBF-SVM is robust with the number of samples we have used in each antibiotic model; this is likely due to the fact that we have used the nested cross validation. According to Vabalas *et al.,* 2019 the nested cross validation approach can give an unbiased performance estimate regardless of the sample size. Vabalas and collaborators indicated that by using a nested cross validation approach the models are able to learn patterns in the data more efficiently and hence can achieve high performance results. In their work, they concluded that separating completely testing and training data is enough to obtain unbiased performance estimates regardless of sample size.

Supplementary Figure 3. Learning Curves. Learning curves of the 21 antibiotics using a wrapper backward selection to evaluate the training and testing performance whilst decreasing the number of samples. (A) The original number of samples for each antibiotic and 5 nested cross validation iterations with an RBF-SVM as the main classifier; (B) SMOTE approach as a pre-processing step to increase the number of samples by adding synthetic samples to the minority class for each antibiotic and 5 nested cross validation iterations with an RBF-SVM as the main classifier. In both cases, the same features used to acquire the performances in the Figure 2 and Supplementary Table 2 are used and kept for all the iterations of the WBS. The red lines indicate the training performance, the green lines the testing performance and the blue vertical line the original number of samples.

We thank the reviewer for his/her suggestion regarding justifying the use of the RBF-SVM in this work and we feel that the analysis we did to address his/her comment improved the quality of the manuscript. We have now updated the manuscript see results lines 266-279 and 309-332 and methods lines 1097-1105 and 1107-1113 and Supplementary Figure 3 and below to describe the analysis done:

Results Lines 266-279: “To verify which classifier performs better out of the 10 classifiers studied we applied a Friedman F-test with Nemenyi post hoc test. With 10 classifiers and 21 antibiotics, the Friedman test is distributed according to the F distribution with 21−1 = 20 and (10−1)×(21−1) = 180 degrees of freedom. The critical value of F(9,180) for α= 0.0001 is 4.0366. For all the metrics studies (AUC, accuracy, sensitivity, specificity, precision and Cohen’s Kappa), the F_F_ statistics null hypothesis was rejected with a confidence level of 99.99%. The null hypothesis states that there are no differences between the average ranks of each classifier over the 21 antibiotics. After, the Nemenyi post-hoc test was performed, and the critical difference diagram was set at 3.407 with a confidence level of 99%. According to Supplementary Figure 2, the classifier RBF-SVM had the best rank for the metrics AUC, accuracy, sensitivity, Cohen’s kappa, second best for precision and third best for specificity. Nonetheless, according to the Nemenyi test, for precision and specificity, RBF-SVM is statistically equivalent when compared to the best ranked classifier in these two metrics. Therefore, the RBF-SVM classifier was selected for this study.”

Results Lines 309-332: “To verify if the number of samples for each antibiotic model was large enough for the test set to be representative, we employed a wrapper backward selection (WBS) in terms of the samples using two different analyses: (i) firstly, we applied the WBS (see Supplementary Figure 3A), to the original pipeline used in this work (i.e. nested cross validation with an RBF-SVM as the classifier and a SMOTE approach during the training phase to balance the classes for the training of the classifier (as used in our ML pipeline), 5 iterations of the nested cross validation were used; (ii) while, for the second analysis, we first performed a SMOTE approach as a pre-processing step to oversample the minority class and then applied the WBS method on the oversampled data, and analysed using 5 iterations of a nested cross validation with a RBF-SVM as the classifier (Supplementary Figure 3B). Supplementary Figure 3A shows that for 4 out of 21 antibiotics (AMP, TET, CFX and MIN) the WBS finished after a few iterations due to the small number of samples in the minority class. Nonetheless, according to the learning curves of the other 17 out of 21 antibiotics on Supplementary Figure 3A the training and test datasets are representative for the initial number of samples in each antibiotic; with a reduced number of samples the majority of the antibiotics have a decrease in the test fold performance, indicating that a reduced number of samples in this case can cause an overfitting of the classifier, since the test fold is not representative anymore. The curve of the test performance for all models is reaching an asymptote indicating that increasing sample sizes further would be unlikely to significantly improve model performance. Moreover, on Supplementary Figure 3B, which creates synthetic data for the minority class during the pre-processing, reveals a similar result to the first approach in Supplementary Figure 3A. The shape of the learning curves is kept similar between the two studies and it confirms for all the antibiotics that an increase in the number of samples would not have a big impact in the performance when compared to the original number of samples.”

Methods Lines 1097-1105: “To compare the results obtained by the 10 different classifiers used, a Friedman Statistical F-test was employed. The Friedman test F_F_ with Iman-Davenport correction (Iman & Davenport, 1980) is employed for statistical comparison of multiple classifiers as suggested by Demsar *et al.* (2006). First, we rank the algorithms for each dataset separately, i.e., the best algorithm gets ranking 1, the second best ranking 2, and so on. In case of ties, average ranks are assigned. Next, we apply the F_F_ and verify if the null hypothesis is rejected. After, the post-hoc Nemenyi test (Demsar *et al.* 2006) was used to find if there is a single classifier or a group of classifiers that differ in terms of their average rank after the F_F_ test has rejected the null hypothesis that the performance of the comparisons on the groups of data is similar.”

Method Lines 1107-1113: “To analyse if the antibiotics studied had enough samples to make the test representative, a wrapper backward selection (WBS) approach in terms of samples was used, i.e. if initially we have 100 samples we are first going to test the model using all 99 possible sample combinations (leaving one out), the worst performance is identified and the sample that was left out on this performance is removed for the next iteration. This is done until the minimum number of samples (12) is reached for one of the classes. This minimum number of samples is a requirement for the SMOTE approach used in the classification framework.”

Beleites, C, et al. "Sample size planning for classification models." Analytica chimica acta 760 (2013): 25-33.

Brownlee, J. (2019) “How to use Learning Curves to Diagnose Machine Learning Model Performance” Machine Learning Mastery. Accessed on January 28^th^ 2022. https://machinelearningmastery.com/learning-curves-for-diagnosing-machine-learning-model-performance/

Demsar, J. Statistical comparisons of classifiers over multiple data sets, J. Mach. Learn. Res., vol. 7, pp. 1–30, Dec. 2006, ISSN: 1532-4435

Iman, R. L. and Davenport, J. M. Approximations of the critical region of the fbietkan statistic, Communications in Statistics - Theory and Methods, vol. 9,no. 6, pp. 571–595, 1980

Liu, Z., et al. (2020). "Evaluation of Machine Learning Models for Predicting Antimicrobial Resistance of Actinobacillus pleuropneumoniae From Whole Genome Sequences." Frontiers in Microbiology 11.

Her, H. L. and Wu Y. W. (2018). "A pan-genome-based machine learning approach for predicting antimicrobial resistance activities of the Escherichia coli strains." Bioinformatics 34(13): i89-i95.

Hyun JC, Kavvas ES, Monk JM, Palsson BO. Machine learning with random subspace ensembles identifies antimicrobial resistance determinants from pan-genomes of three pathogens. PLOS Computational Biology. 2020;16(3):e1007608.

Maguire, F., et al. (2019). "Identification of Primary Antimicrobial Resistance Drivers in Agricultural Nontyphoidal Salmonella enterica Serovars by Using Machine Learning." mSystems 4(4): e00211-00219.

Moradigaravand D, Palm M, Farewell A, Mustonen V, Warringer J, Parts L. Prediction of antibiotic resistance in Escherichia coli from large-scale pan-genome data. Plos Computational Biology. 2018;14(12):e1006258.

Pappu, V., & Pardalos, P. M. (2014). High-dimensional data classification. In Clusters, orders, and trees: methods and applications (pp. 119-150). Springer, New York, NY.

Pincus, N. B., et al. (2020). "A Genome-Based Model to Predict the Virulence of Pseudomonas aeruginosa Isolates." MBio 11(4): e01527-01520.

Ren Y, Chakraborty T, Doijad S, Falgenhauer L, Falgenhauer J, Goesmann A, et al. Prediction of antimicrobial resistance based on whole-genome sequencing and machine learning. Bioinformatics (Oxford, England). 2021;38(2):325-34.

Vabalas A, Gowen E, Poliakoff E, Casson AJ (2019) “Machine learning algorithm validation with a limited sample size”. PLOS ONE 14(11): e0224365.

**Response to data availability**

**Have the authors made all data and (if applicable) computational code underlying the findings in their manuscript fully available?**

The [PLOS Data policy](https://journals.plos.org/ploscompbiol/s/materials-and-software-sharing) requires authors to make all data and code underlying the findings described in their manuscript fully available without restriction, with rare exception (please refer to the Data Availability Statement in the manuscript PDF file). The data and code should be provided as part of the manuscript or its supporting information, or deposited to a public repository. For example, in addition to summary statistics, the data points behind means, medians and variance measures should be available. If there are restrictions on publicly sharing data or code —e.g. participant privacy or use of data from a third party—those must be specified.

Reviewer #1: **No:** Only code is shared, no data.

Reviewer #2: Yes

We apologise that the Reviewer 1 was unable to find the data sharing link in the Data and Code Availability section of the Manuscript metadata. All the information was submitted initially on the manuscript online form as requested by the submission guidelines from PLOS Computational Biology (https://journals.plos.org/ploscompbiol/s/submission-guidelines). Nonetheless, we have now added this information (code and data availability statements) as well as competing interests and funding statements, into the main manuscript file for clarity. See lines 1201-1220 and below:

“Data Availability

Short-read sequence data for all 154 isolates used in this study are deposited in the NCBI SRA and can be found associated with BioProject PRJNA675772 available on: <https://dataview.ncbi.nlm.nih.gov/object/PRJNA675772?reviewer=jl6fn04hrb5iof28ulejqp857p>.

Computer code

For the reviewing process, the code is temporarily available at the following Dropbox link: link: <https://www.dropbox.com/sh/jkud4xiqllcmoes/AADw3u-QhAxU-eCPIOtwnJcla?dl=0>

(the code will be made publicly available on GitHub upon publication).

Competing interests

The authors declare that they have no competing interests.

Funding

This work was supported by the Ministry of Science and Technology of P. R. China under Grant Key Project of International Scientific and Technological Innovation Cooperation Between Governments (number 2018YFE0101500) and the InnovateUK grant [104986], FARMWATCH: Fight AbR with Machine learning and a Wide Array of sensing TeCHnologies.”

**Additional Edits**

All manuscript figures have been edited to meet the font and figure size requirements from PLoS.

All figure panel labels have been capitalised and the manuscript changed accordingly.
